# Supplementary material for: VTA monosynaptic connections by local glutamate and GABA neurons and their distinct roles in behavior
Source: Nat Commun. 2025 Sep 26;16:8500. doi: 10.1038/s41467-025-63396-0 (PMC12475231; doi:10.1038/s41467-025-63396-0)
Supplement: Supplementary file 1 — Supplementary Information [file 41467_2025_63396_MOESM1_ESM.pdf]

Nature Communications

Supplementary information

VTA monosynaptic connections by local glutamate and GABA neurons and their distinct roles in behavior

M. Flavia Barbano, Huiling Wang, Shiliang Zhang, Alexey V. Shevelkin, Kevin J. Yu, Christopher T. Richie, Bing Liu, Suyun Hahn, Rong Ye, Marisela Morales

Supplementary Figures

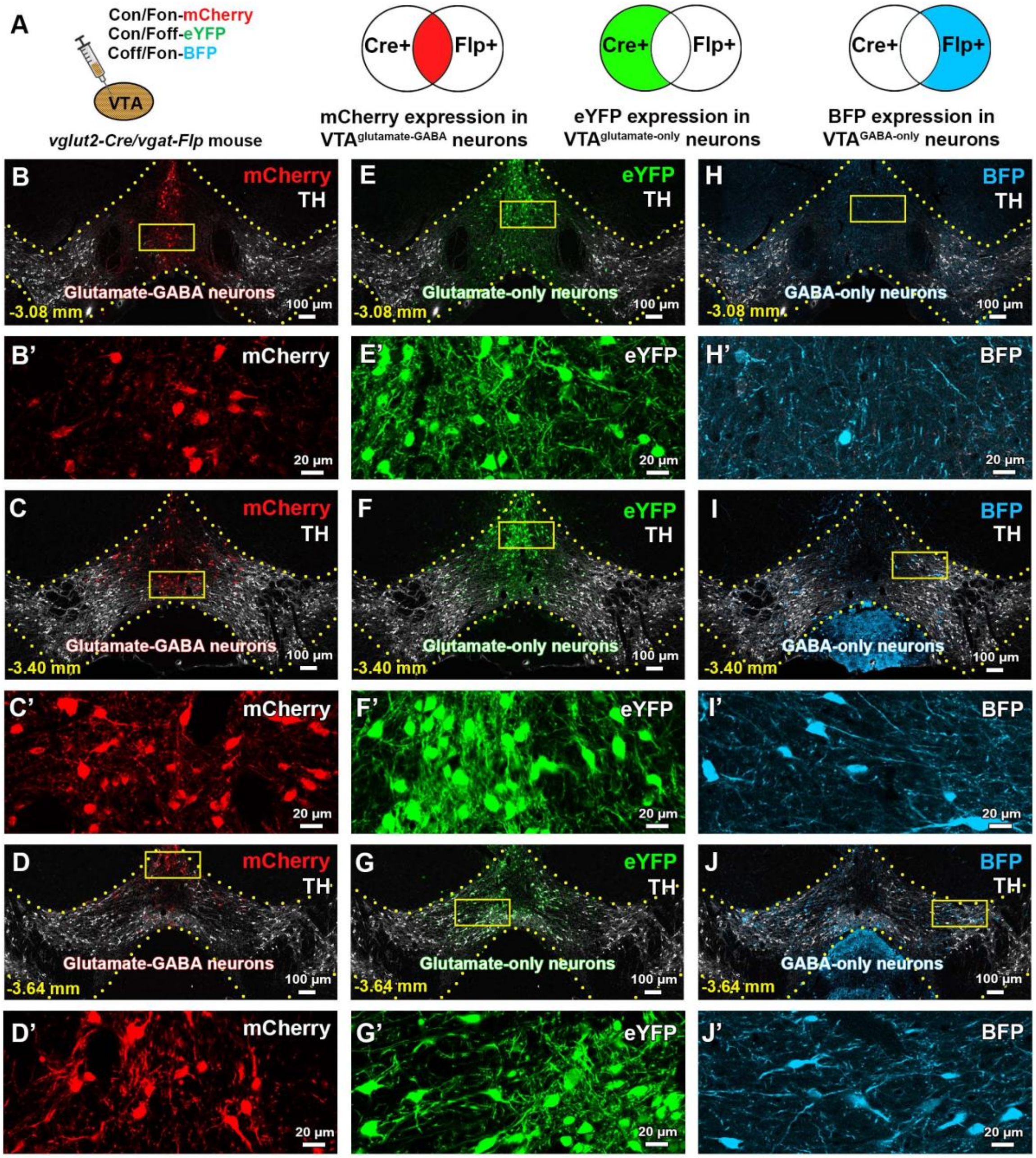

Supplementary figure 1. Distribution of transfected $VTA^{glutamate\text{-}GABA}$, $VTA^{glutamate\text{-}only}$ and $VTA^{GABA\text{-}only}$ neurons. A. Injection of a cocktail of INTRSECT2.0 vectors (AAV-Con/Fon-mCherry, AAV-Con/Foff-eYFP, AAV-Coff/Fon-BFP) into the VTA of *vglut2-Cre/vgat-Flp* mice. **B-D'**. Rostro-caudal detection of transfected $VTA^{glutamate\text{-}GABA}$ neurons expressing mCherry [bregma -3.08 mm (B), bregma -3.40 mm (C) and bregma -3.64 mm (D)]. Rectangles in B, C, and D are seen at higher magnification in B', C', and D'. **E-G'**. Rostro-caudal detection of transfected $VTA^{glutamate\text{-}only}$ neurons expressing eYFP [bregma -3.08 mm (E), bregma -3.40 mm (F) and bregma -3.64 mm (G)]. Rectangles in E, F, and G are seen at higher magnification in E', F', and G'. **H-J'**. Rostro-caudal detection of transfected $VTA^{GABA\text{-}only}$ neurons expressing BFP [bregma -3.08 mm (H), bregma -3.40 mm (I) and bregma -3.64 mm (J)]. Rectangles in H, I, and J are seen at higher magnification in H', I', and J'.

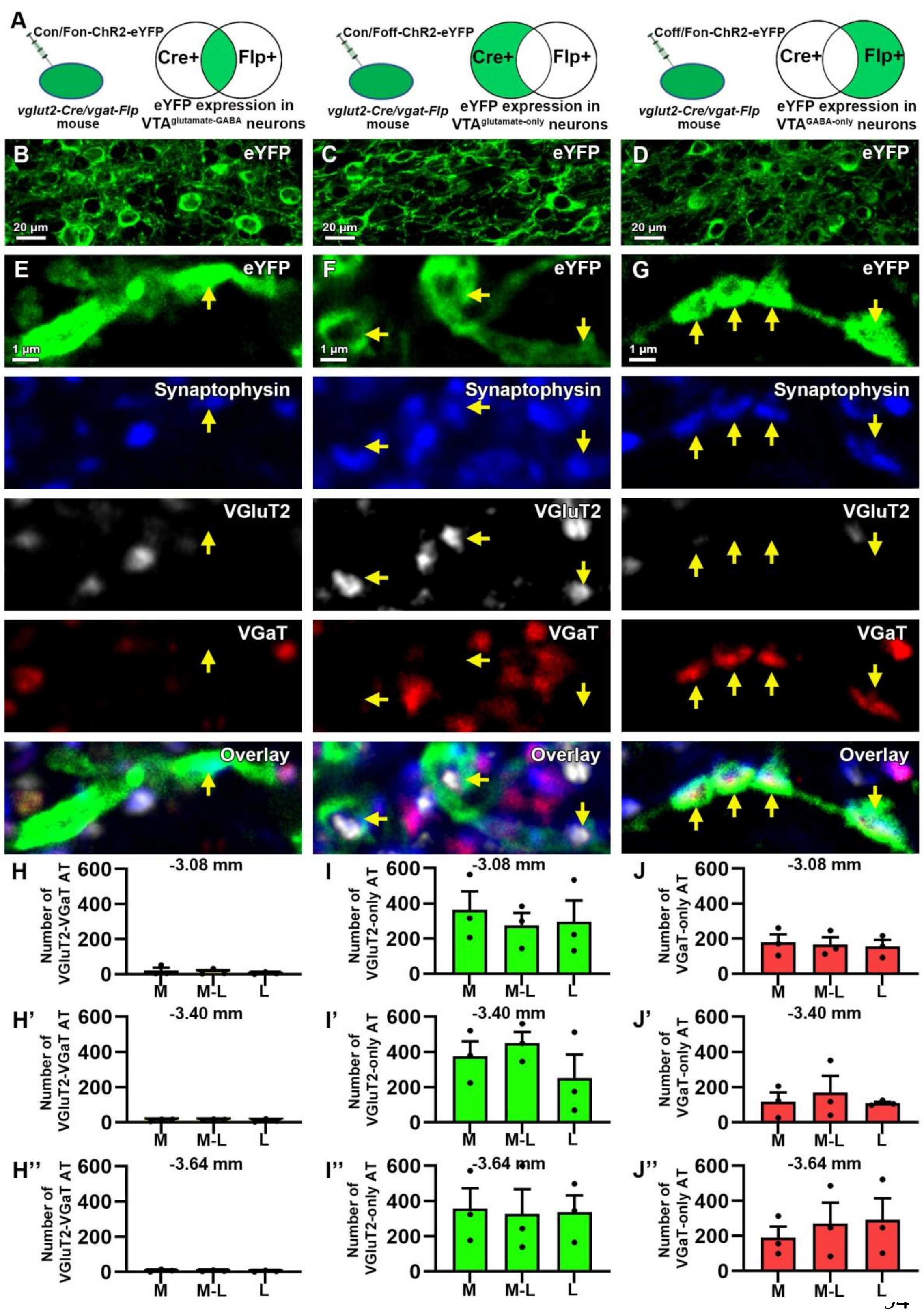

Supplementary figure 2. VTA distribution of axon terminals from VTA$^{\text{glutamate-GABA}}$, VTA$^{\text{glutamate-only}}$ and VTA$^{\text{GABA-only}}$ neurons. A. Intra-VTA injections of AAV-Con/Fon-ChR2-eYFP viral vector to drive eYFP expression in VTA$^{\text{glutamate-GABA}}$ neurons, AAV-Con/Foff-ChR2-eYFP viral vector to drive eYFP expression in VTA$^{\text{glutamate-only}}$ neurons, or AAV-Coff/Fon-ChR2-eYFP viral vector to drive eYFP expression in VTA$^{\text{GABA-only}}$ neurons. **B-D.** Detection of eYFP signals in the transfected cell bodies of VTA$^{\text{glutamate-GABA}}$ neurons (B), VTA$^{\text{glutamate-only}}$ neurons (C), or VTA$^{\text{GABA-only}}$ neurons (D). **E-G.** Detection of eYFP axons (green) and corresponding axon terminals (ATs) identified by immunodetection of synaptophysin (blue), VGluT2 (white) or VGaT (red). **E.** ATs from VTA$^{\text{glutamate-GABA}}$ neurons co-expressing eYFP and synaptophysin, but lacking VGluT2 and VGaT are indicated by arrows. **F.** ATs from VTA$^{\text{glutamate-only}}$ neurons co-expressing eYFP, synaptophysin and VGluT2, but lacking VGaT are indicated by arrows. **G.** ATs from VTA$^{\text{GABA-only}}$ neurons co-expressing eYFP, synaptophysin and VGaT, but lacking VGluT2 are indicated by arrows. **H-H''.** VTA rostro-caudal distribution of VGluT2-VGaT axon terminals [bregma -3.08 mm (H), bregma -3.40 mm (H') and bregma -3.64 mm (H'')]. **I-I''.** VTA rostro-caudal distribution of VGluT2-only axon terminals [bregma -3.08 mm (I), bregma -3.40 mm (I') and bregma -3.64 mm (I'')]. **J-J''.** VTA rostrocaudal distribution of VGaT-only axon terminals [bregma -3.08 mm (J), bregma -3.40 mm (J') and bregma -3.64 mm (J'')]. M, medial VTA; M-L, mediolateral VTA; L, lateral VTA. Data are shown as mean ± SEM. Source data are provided as a Source Data file.

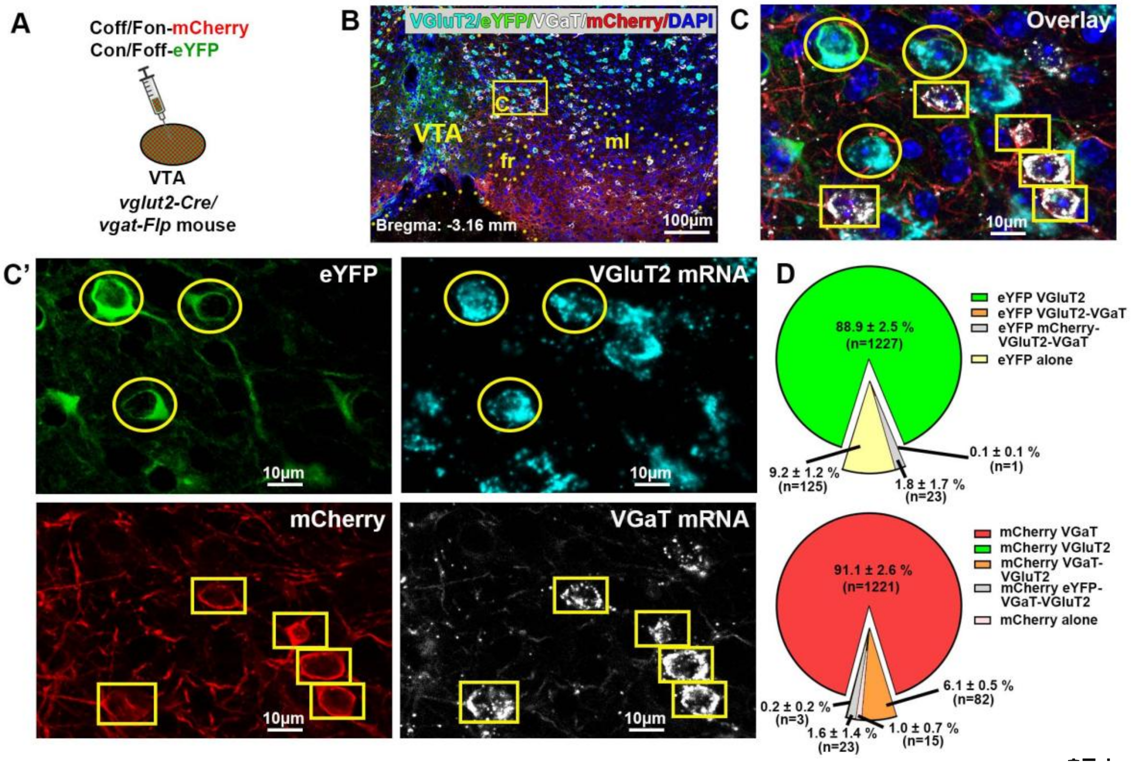

Supplementary figure 3. Simultaneous targeting of $\text{VTA}^{\text{glutamate-only}}$ and $\text{VTA}^{\text{GABA-only}}$ neurons. A. Intra-VTA injections of a cocktail of Con/Foff-eYFP (for the expression of eYFP in $\text{VTA}^{\text{glutamate-only}}$ neurons) and Coff/Fon-mCherry (for the expression of mCherry in $\text{VTA}^{\text{GABA-only}}$ neurons) viral vectors in *vglut2-Cre/vgat-Flp* mice. **B.** Confocal micrograph of VTA at low magnification. **C.** High magnification of square area in B showing VTA eYFP neurons (green neurons delineated by yellow circles) intermingled with VTA mCherry neurons (red neurons delineated by yellow squares). **C'.** VTA neuronal co-expression of eYFP and VGluT2 mRNA (cyan) and neuronal co-expression of mCherry and VGaT mRNA (white). **D.** Detection of VGluT2 mRNAs within the subpopulations of VTA neurons expressing eYFP (top) and detection of VGaT mRNAs within the subpopulations of VTA neurons expressing mCherry (bottom). Data are shown as mean ± SEM. The number of total counted neurons ("n") is shown in each pie graph (3 mice/group). fr, fasciculus retroflexus; ml, medial lemniscus; VTA, ventral tegmental area. Source data are provided as a Source Data file.

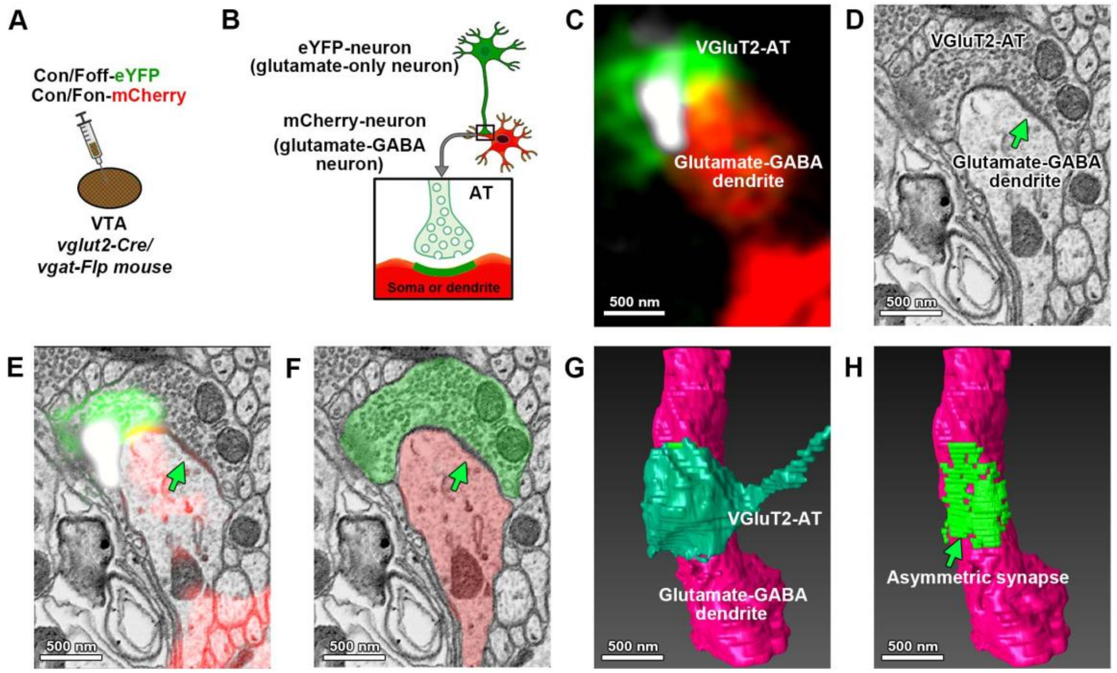

Supplementary figure 4. Tridimensional ultrastructure of synapses between ATs from VTA$^{glutamate\text{-}only}$ neurons and postsynaptic VTA$^{glutamate\text{-}GABA}$ neurons using Correlative Light and Electron Microscopy (CLEM). A. Intra-VTA injection of a cocktail of Con/Foff-eYFP (for the expression of eYFP in VTA$^{glutamate\text{-}only}$ neurons) and Con/Fon-mCherry (for the expression of mCherry in VTA$^{glutamate\text{-}GABA}$ neurons) viral vectors in *vglut2-Cre/vgat-Flp* mice. **B.** Diagram of an asymmetric synapse (created with Motifolio and BioRender [Zhang, S. (2025) https://BioRender.com/hksfm4r]) between an AT from a VTA$^{glutamate\text{-}only}$ neuron (expressing eYFP) and a postsynaptic VTA$^{glutamate\text{-}GABA}$ neuron (expressing mCherry). **C.** VTA confocal micrograph showing one image-frame of an AT out of serial Z-stacks for simultaneous fluorescent detection of three proteins: eYFP (green), VGluT2 (white), and mCherry (red). **D.** Corresponding scanning electron micrograph of the AT imaged by confocal microscopy showing the AT with synaptic vesicles establishing an asymmetric synapse (green arrow) on a VTA$^{glutamate\text{-}GABA}$ dendrite. **E.** Correlation of fluorescent and scanning electron microscopic images showing an asymmetric synapse (green arrow) between the AT from a VTA$^{glutamate\text{-}only}$ neuron [VGluT2-AT, co-expressing eYFP (green) and VGluT2 (white)] and the postsynaptic dendrite from a VTA$^{glutamate\text{-}GABA}$ neuron (expressing mCherry). **F.** Segmentation of scanning electron microscopic image showing an asymmetric synapse (green arrow) between the AT from a VTA$^{glutamate\text{-}only}$ neuron (green) and the postsynaptic dendrite from a VTA$^{glutamate\text{-}GABA}$ neuron (red). **G-H.** Tridimensional ultrastructural reconstruction of VTA local circuitry from serial scanning electron microscopic images showing an AT from a VTA$^{glutamate\text{-}only}$ neuron (in G) establishing an asymmetric synapse (green arrow in H) on a postsynaptic VTA$^{glutamate\text{-}GABA}$ neuron.

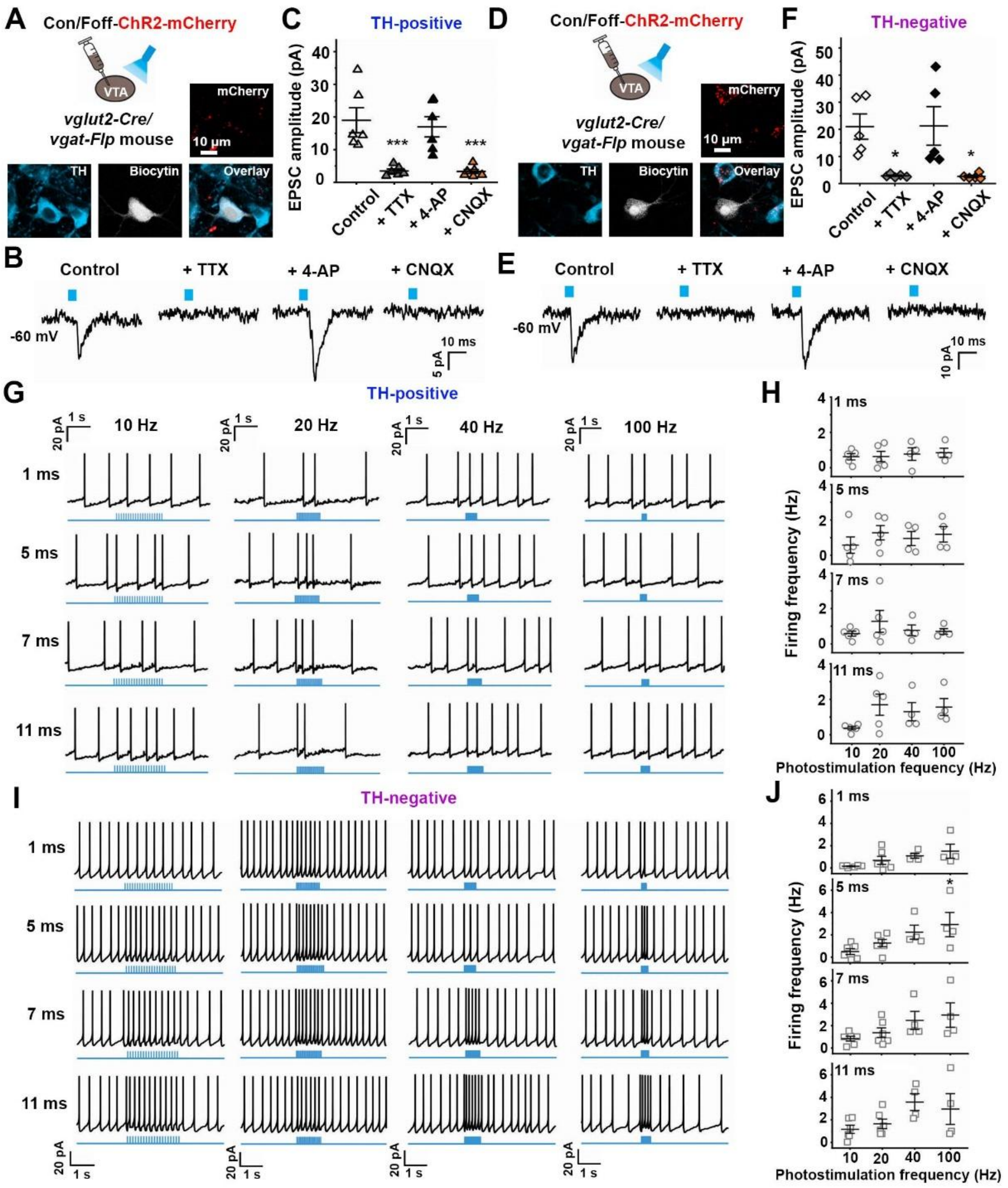

Supplementary figure 5. VTA electrophysiological properties of the monosynaptic connections of VTA$^{glutamate\text{-}only}$ neurons. **A.** Injection of Con/Foff-ChR2-mCherry viral vector into the VTA of *vglut2-Cre/vgat-Flp* mice. Brain coronal section containing VTA biocytin-filled cells (white) expressing TH (cyan) and surrounded by mCherry fibers. **B.** Traces from a TH-positive neuron in response to VTA photostimulation obtained before (control), and after sequential application of TTX (0.5 µM), 4-AP (200 µM), and CNQX (10 µM). Blue squares indicate light stimulation (5 ms). **C.** EPSC amplitude (pA) in TH-positive neurons under different conditions [control (aCSF; 18.98 ± 3.83 pA), + TTX (3.47 ± 0.6 pA), + 4-AP (16.98 ± 3.09 pA), + CNQX (3.32 ± 0.65 pA)] were significantly different ($F_{(3,15)} = 17.99$; $p = 0.002$; n = 6 neurons from 3 mice). **D.** Injection of Con/Foff-ChR2-mCherry viral vector into the VTA of *vglut2-Cre/vgat-Flp* mice. Brain coronal section containing VTA biocytin-filled cells (white) without TH and surrounded by mCherry fibers. **E.** Traces from a TH-negative neuron in response to VTA photostimulation obtained before (control), and after sequential application of TTX, 4-AP, and CNQX. **F.** EPSC amplitude (pA) in TH-negative neurons under different conditions [control (aCSF; 21 ± 4.68 pA), + TTX (2.86 ± 0.25 pA), + 4-AP (21.24 ± 7.11 pA), + CNQX (2.62 ± 0.44 pA)] were significantly different ($F_{(3,12)} = 9.02$; $p = 0.04$; n = 5 neurons from 4 mice). **G.** Traces from a TH-positive neuron in response to VTA photostimulation for 1, 5, 7, or 11 ms, at different frequencies (10, 20, 40, 100 Hz). **H.** Neuronal firing frequency (Hz) in response to varying photosimulation frequencies at different durations of photostimulation in TH-positive neurons (n = 5 from 4 mice; 1 ms: $F_{(3,9)} = 0.30$; $p = 0.82$; 5 ms: $F_{(3,9)} = 0.26$; $p = 0.85$; 7 ms: $F_{(3,9)} = 0.36$; $p = 0.78$; 11 ms: $F_{(3,9)} = 1.01$; $p = 0.43$). **I.** Traces from a TH-negative neuron in response to VTA photostimulation for 1, 5, 7, or 11 ms, at different frequencies (10, 20, 40, 100 Hz). **J.** Neuronal firing frequency (Hz) in response to varying photosimulation frequencies at different durations of photostimulation in TH-negative neurons (n = 6 from 5 mice; 1 ms: $F_{(3,9)} = 1.57$; $p = 0.26$; 5 ms: $F_{(3,9)} = 4.26$; $p = 0.05$; 7 ms: $F_{(3,9)} = 1.31$; $p = 0.34$; 11 ms: $F_{(3,9)} = 1.28$; $p = 0.34$). Data are shown as mean ± SEM. One-way ANOVA with Tukey HSD post hoc test. * $p < 0.05$, *** $p < 0.001$, against control condition (C, F) or 10 Hz photostimulation group (J). Source data are provided as a Source Data file.

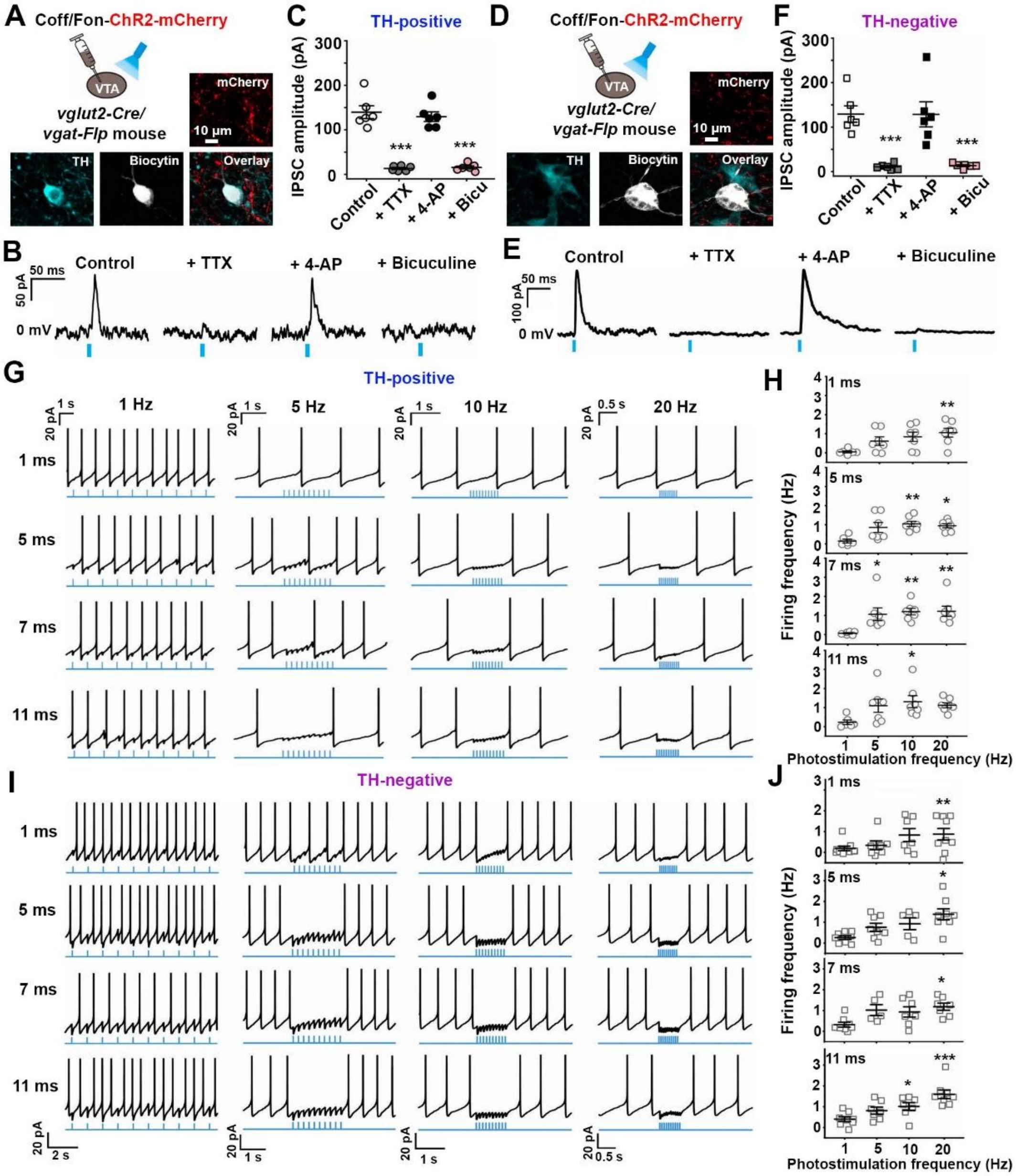

Supplementary figure 6. VTA electrophysiological properties of monosynaptic connection of VTA$^{GABA\text{-}only}$ neurons. A. Injection of Coff/Fon-ChR2-mCherry viral vector into the VTA of *vglut2-Cre/vgat-Flp* mice. Brain coronal section containing VTA biocytin-filled cells (white) expressing TH (cyan) and surrounded by mCherry fibers. **B.** Traces from a TH-positive neuron in response to VTA photostimulation obtained before (control), and after sequential application of TTX (0.5 µM), 4-AP (200 µM), and bicuculine (10 µM). Blue squares indicate light stimulation (5 ms). **C.** IPSC amplitude (pA) in TH-positive neurons under different conditions [control (aCSF; 139.58 ± 14.36 pA), + TTX (13.27 ± 2.35 pA), + 4-AP (129.62 ± 10.76 pA), + bicuculine (15.72 ± 3.25 pA)] were significantly different ($F_{(3,15)} = 63.11$; $p < 0.00001$; n = 6 neurons from 5 mice). **D.** Viral injection of Con/Foff-eYFP and Coff/Fon-ChR2-mCherry into the VTA of *vglut2-Cre/vgat-Flp* mice. Brain coronal section containing VTA biocytin-filled cells (white) without TH and surrounded by mCherry fibers. **E.** Traces from a TH-negative neuron in response to VTA photostimulation obtained before (control), and after sequential application of TTX, 4-AP, and bicuculine. **F.** IPSC amplitude (pA) in TH-negative neurons under different conditions [control (aCSF; 129.28 ± 18.28 pA), + TTX (10.8 ± 2.63 pA), + 4-AP (128.63 ± 28.12 pA), + bicuculine (13.37 ± 2.07 pA)] were significantly different ($F_{(3,15)} = 23.28$; $p = 0.004$; n = 6 neurons from 5 mice). **G.** Traces from a TH-positive neuron in response to VTA photostimulation for 1, 5, 7, and 11 ms, at different frequencies (1, 5, 10, 20 Hz). **H.** Neuronal firing frequency (Hz) in response to varying photosimulation frequencies at different durations of photostimulation in TH-positive neurons (n = 7 from 5 mice; 1 ms: $F_{(3,15)} = 7.49$; $p = 0.003$; 5 ms: $F_{(3,15)} = 5.30$; $p = 0.04$; 7 ms: $F_{(3,15)} = 10.92$; $p = 0.01$; 11 ms: $F_{(3,15)} = 6.24$; $p = 0.02$). **I.** Traces from a TH-negative neuron in response to VTA photostimulation for 1, 5, 7, and 11 ms, at different frequencies (1, 5, 10, 20 Hz). **J.** Neuronal firing frequency (Hz) in response to varying photosimulation frequencies at different durations of photostimulation in TH-negative neurons (n = 8 from 5 mice; 1 ms: $F_{(3,21)} = 5.16$; $p = 0.008$; 5 ms: $F_{(3,21)} = 4.53$; $p = 0.01$; 7 ms: $F_{(3,21)} = 3.25$; $p = 0.04$; 11 ms: $F_{(3,21)} = 6.04$; $p = 0.02$). Data are shown as mean ± SEM. One-way ANOVA with Tukey HSD post hoc test. * $p < 0.05$, ** $p < 0.01$, ***$p < 0.001$, against control condition (C, F) or 1 Hz photostimulation group (H, J). Source data are provided as a Source Data file.

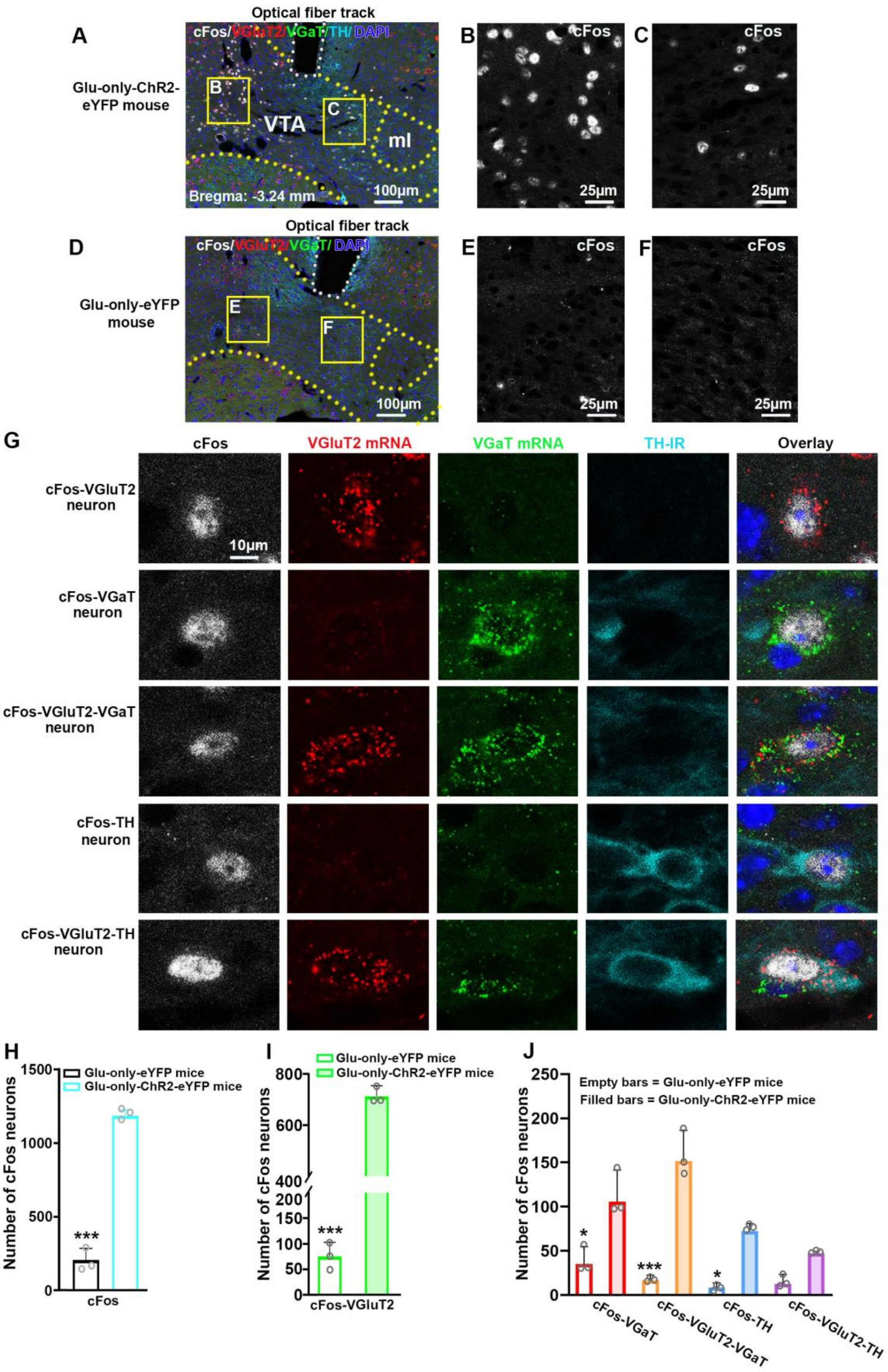

Supplementary figure 7. Photostimulation of VTA$^{glutamate\text{-}only}$ neurons induces cFos expression in different subpopulations of VTA neurons. **A-F.** cFos expression (white nuclei) induced by VTA photostimulation of VTA$^{glutamate\text{-}only}$ neurons in Glu-only-ChR2-eYFP (A-C) or Glu-only-eYFP control mice (D-F). **B-C.** Boxes in A at higher magnification showing cFos expression. **E-F.** Boxes in D at higher magnification showing cFos expression. **G.** VTA neurons expressing cFos and co-expressing VGluT2 mRNA, VGaT mRNA, TH protein or their combination. **H.** The number of VTA cFos neurons was higher in Glu-only-ChR2-eYFP mice (1181.00 ± 23.79 neurons; n = 3 mice, 15 sections/mouse) than in Glu-only-eYFP control mice (203.33 ± 44.98 neurons; n = 3 mice, 15 section/mouse; $t_{(4)}$ = 19.64, p = 0.00004, two-tailed t test). **I.** The number of VTA cFos neurons co-expressing VGluT2 mRNA was higher in Glu-only-ChR2-eYFP mice than in Glu-only-eYFP control mice, ($t_{(4)}$ = 22.31, p = 0.00002, two-tailed t test). **J.** The number of VTA cFos neurons co-expressing VGaT mRNA, VGluT2 and VGaT mRNAs or TH immunoreactivity was higher in Glu-only-ChR2-eYFP mice than in Glu-only-eYFP control mice (group × phenotype: $F_{(3,12)}$ = 6.15, p = 0.009, ANOVA with Tukey HSD post hoc test). Data are shown as mean ± SEM. * p < 0.05, ** p < 0.01, *** p < 0.001, against Glu-only-ChR2-eYFP mice. Source data are provided as a Source Data file.

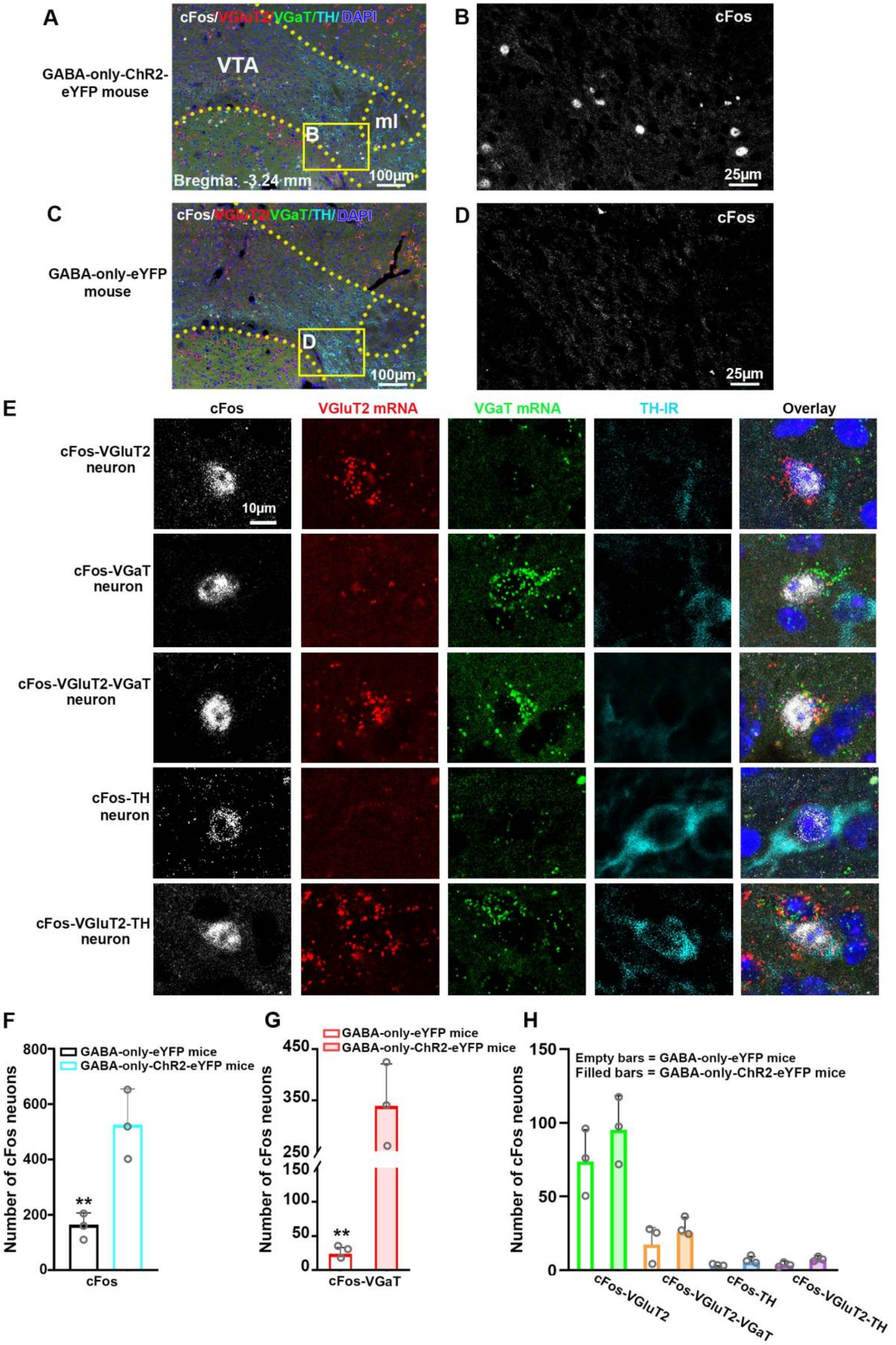

Supplementary figure 8. Photostimulation of VTA$^{\text{GABA-only}}$ neurons induces cFos expression mostly in VTA$^{\text{GABA-only}}$ neurons. **A-D**. cFos expression (white nuclei) induced by VTA photostimulation of VTA$^{\text{GABA-only}}$ neurons in GABA-only-ChR2-eYFP mice (A-B) or GABA-only-eYFP control mice (C-D). **B.** Box in A at higher magnification showing cFos-expressing neurons. **D.** Box in C at higher magnification showing cFos-expressing neurons. **E.** VTA cFos neurons co-expressing VGluT2 mRNA, VGaT mRNA, TH protein or their combination. **F.** The number of VTA neurons expressing cFos was higher in GABA-only-ChR2-eYFP mice (525.00 ± 74.58 neurons; n = 3 mice, 15 sections/mouse) than in GABA-only-eYFP control mice (162.67 ± 24.54 neurons; n = 3 mice, 15 section/mouse; $t_{(4)} = 4.62$, p = 0.01, two-tailed t test). **G**. The number of VTA cFos neurons co-expressing VGaT mRNA was higher in GABA-only-ChR2-eYFP mice than in GABA-only-eYFP control mice ($t_{(4)} = 6.57$, p = 0.003, two-tailed t test). **H.** The number of cFos neurons co-expressing VGluT2 mRNA, both VGluT2 and VGaT mRNAs, TH immunoreactivity, or VGluT2 mRNA and TH immunoreactivity was similar between both groups of mice (group × phenotype: $F_{(3,12)} = 1.29$, p = 0.25, ANOVA with Tukey HSD post hoc test). Data are shown as mean ± SEM. ** p < 0.01, *** p < 0.001, against GABA-only-ChR2-eYFP mice. Source data are provided as a Source Data file.

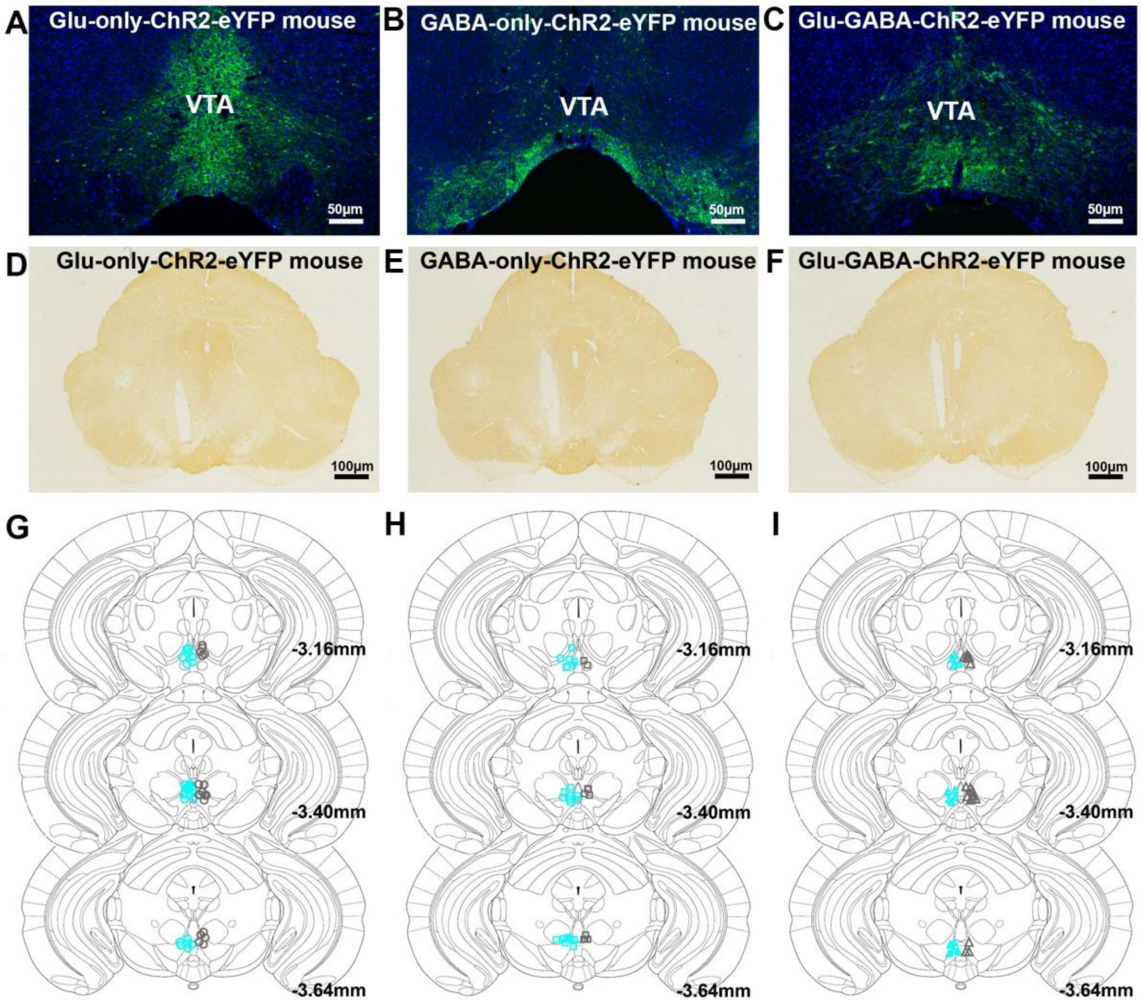

Supplementary figure 9. Viral expression and optic fiber placements in the VTA. **A-C.** Detection of eYFP expression in the VTA of a Glu-only-ChR2-eYFP mouse (A), a GABA-only-ChR2-eYFP mouse (B), and a Glu-GABA-ChR2-eYFP mouse (C). **D-F.** Optic fiber track in the VTA of a Glu-only-ChR2-eYFP mouse (D), a GABA-only-ChR2-eYFP mouse (E), and a Glu-GABA-ChR2-eYFP mouse (F). **G-I.** Optic fiber tip placements in the VTA of mice used for behavioral studies. **G**. Optic fiber tips in the VTA of Glu-only-ChR2-eYFP mice (n = 24, blue circles) and Glu-only-eYFP mice (n = 18, grey circles). **H**. Optical fiber tips in the VTA of GABA-only-ChR2-eYFP mice (n = 19, blue squares) and GABA-only-eYFP mice (n = 10, grey squares). **I**. Optical fiber tips in the VTA of Glu-GABA-ChR2-eYFP mice (n = 14, blue triangles) and Glu-GABA-eYFP mice (n = 16, grey triangles).

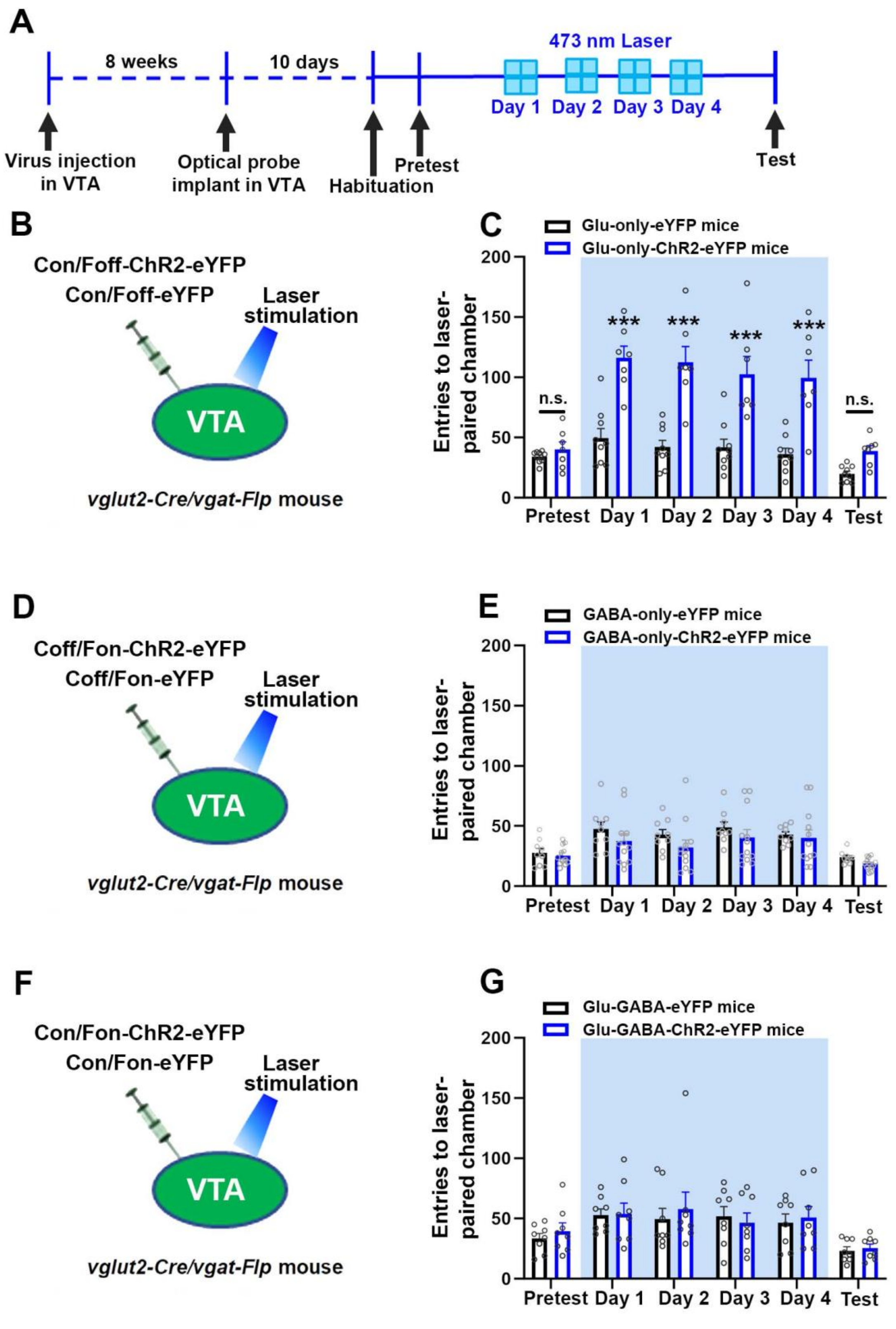

Supplementary figure 10. Photostimulation of $\text{VTA}^{\text{glutamate-only}}$ neurons increases entries to the laser-paired chamber, whereas photostimulation of $\text{VTA}^{\text{GABA-only}}$ or dual $\text{VTA}^{\text{glutamate-GABA}}$ neurons does not affect entries to the laser-paired chamber. A. Timeline for behavioral testing. **B.** VTA injection of Con/Foff viral vectors in *vglut2-Cre/vgat-Flp* mice (to target $\text{VTA}^{\text{glutamate-only}}$ neurons) and VTA photostimulation. **C.** Glu-only-ChR2-eYFP mice (n = 7), but not Glu-only-eYFP control mice (n = 9), showed increases in entries to the laser-paired chamber during the photostimulation sessions but not during the pretest or test sessions without photostimulation (group x day: $F_{(5,70)}$ = 12.59; p = 0.00001). **D.** VTA injection of Coff/Fon viral vectors in *vglut2-Cre/vgat-Flp* mice (to target $\text{VTA}^{\text{GABA-only}}$ neurons) and VTA photostimulation. **E.** The number of entries to the laser-paired chamber was similar between GABA-only-ChR2-eYFP (n = 12) and GABA-only-eYFP control mice (n = 9; group x day: $F_{(5,95)}$ = 0.40; p = 0.85). **F.** VTA injection of Con/Fon viral vectors in *vglut2-Cre/vgat-Flp* mice (to target $\text{VTA}^{\text{glutamate-GABA}}$ neurons) and VTA photostimulation. **G.** The number of entries to the laser-paired chamber was similar between Glu-GABA-ChR2-eYFP (n = 8) and Glu-GABA-eYFP control mice (n = 8; $F_{(5,70)}$ = 0.45; p = 0.81). Data are shown as mean ± SEM. Two-way ANOVA with Tukey HSD post hoc test. *** p < 0.001 against Glu-only-eYFP mice. Source data are provided as a Source Data file.

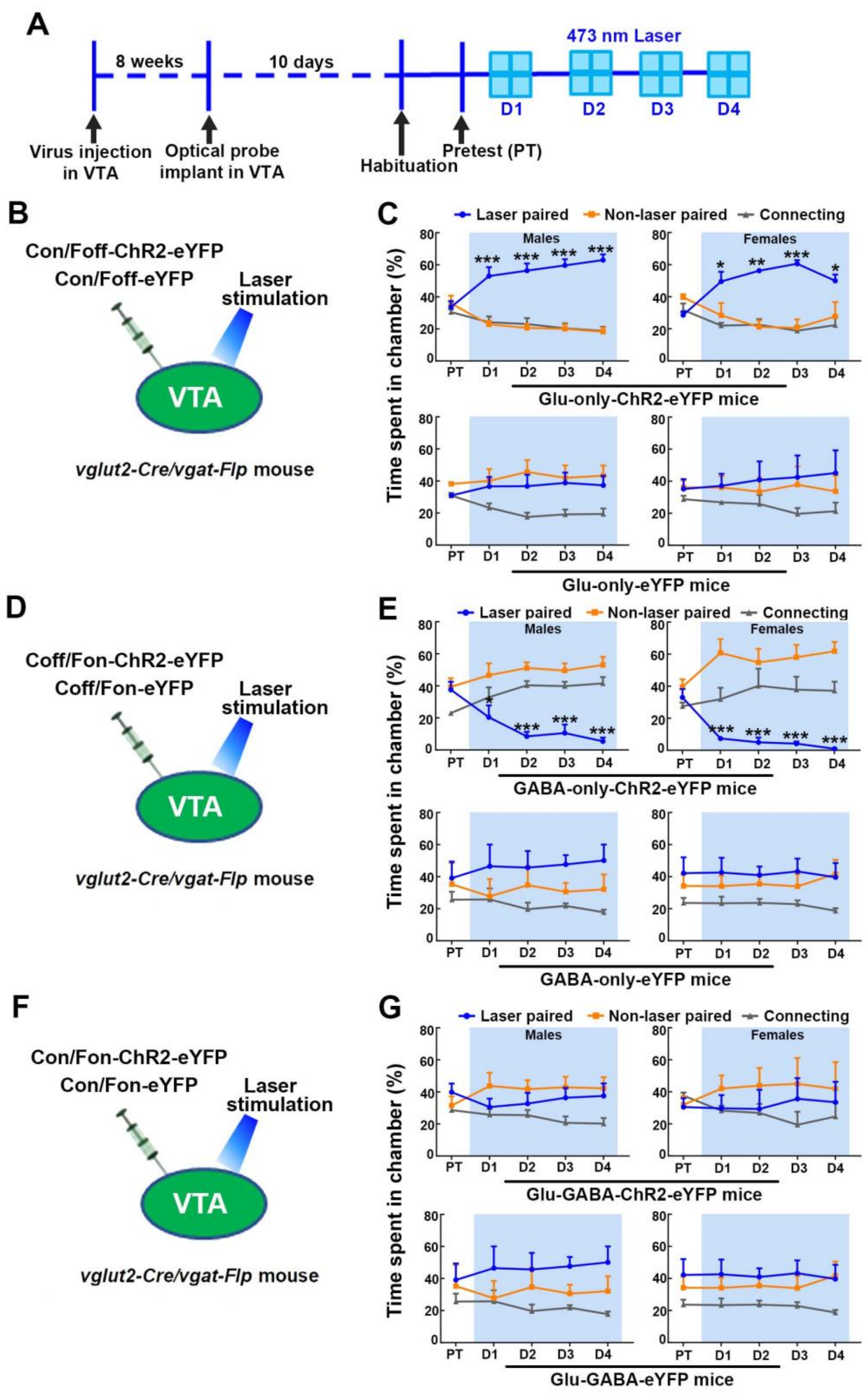

Supplementary figure 11. Photostimulation of VTA$^{\text{glutamate-only}}$, VTA$^{\text{GABA-only}}$ or dual VTA$^{\text{glutamate-GABA}}$ neurons drives the same behaviors in male and female mice. **A.** Timeline for behavioral testing. **B.** VTA injection of Con/Foff viral vectors in *vglut2-Cre/vgat-Flp* mice (to target VTA$^{\text{glutamate-only}}$ neurons) and VTA photostimulation. **C.** Male (n = 5) and female (n = 2) Glu-only-ChR2-eYFP, but not male (n = 5) and female (n = 4) Glu-only-eYFP control mice, spent significantly more time in the photostimulation-paired chamber during the photostimulation sessions (male Glu-only-ChR2-eYFP mice: day x chamber: $F_{(8,32)} = 8.24$; p = 0.00001; female Glu-only-ChR2-eYFP mice: day x chamber: $F_{(8,8)} = 12.19$; p = 0.001; male Glu-only-eYFP mice: day x chamber: $F_{(8,32)} = 1.56$; p = 0.18; female Glu-only-eYFP mice: day x chamber: $F_{(8,24)} = 0.49$; p = 0.85). **D.** VTA injection of Coff/Fon viral vectors in *vglut2-Cre/vgat-Flp* mice (to target VTA$^{\text{GABA-only}}$ neurons) and VTA photostimulation. **E.** GABA-only-ChR2-eYFP male (n = 8) and female (n = 4), but not GABA-only-eYFP control male (n = 4) and female (n = 5) mice, spent significantly less time in the photostimulation-paired chamber during the photostimulation sessions (male GABA-only-ChR2-eYFP mice: day x chamber: $F_{(8,56)} = 4.72$; p = 0.0002; female GABA-only-ChR2-eYFP mice: day x chamber: $F_{(8,24)} = 4.59$; p = 0.002; male GABA-only-eYFP mice: day x chamber: $F_{(8,24)} = 0.93$; p = 0.51; female GABA-only-eYFP mice: day x chamber: $F_{(8,32)} = 0.94$; p = 0.50). **F.** VTA injection of Con/Fon viral vectors in *vglut2-Cre/vgat-Flp* mice (to target VTA$^{\text{glutamate-GABA}}$ neurons) and VTA photostimulation. **G.** Glu-GABA-ChR2-eYFP male (n = 5) and female (n = 3) mice, as well as Glu-GABA-eYFP control male (n = 5) and female (n = 3) mice spent similar time in the photostimulation-paired and non-paired chambers during the photostimulation sessions (male Glu-GABA-ChR2-eYFP mice: day x chamber: $F_{(8,32)} = 1.39$; p = 0.24; female Glu-GABA-ChR2-eYFP mice: day x chamber: $F_{(8,16)} = 1.12$; p = 0.39; male Glu-GABA-eYFP mice: day x chamber: $F_{(8,32)} = 1.63$; p = 0.16; female Glu-GABA-eYFP mice: day x chamber: $F_{(8,16)} = 1.07$; p = 0.43). Data are shown as mean ± SEM. Two-way ANOVA with Tukey HSD post hoc test. *p < 0.05, **p < 0.01, *** p < 0.001, against non-laser paired chamber. Source data are provided as a Source Data file.

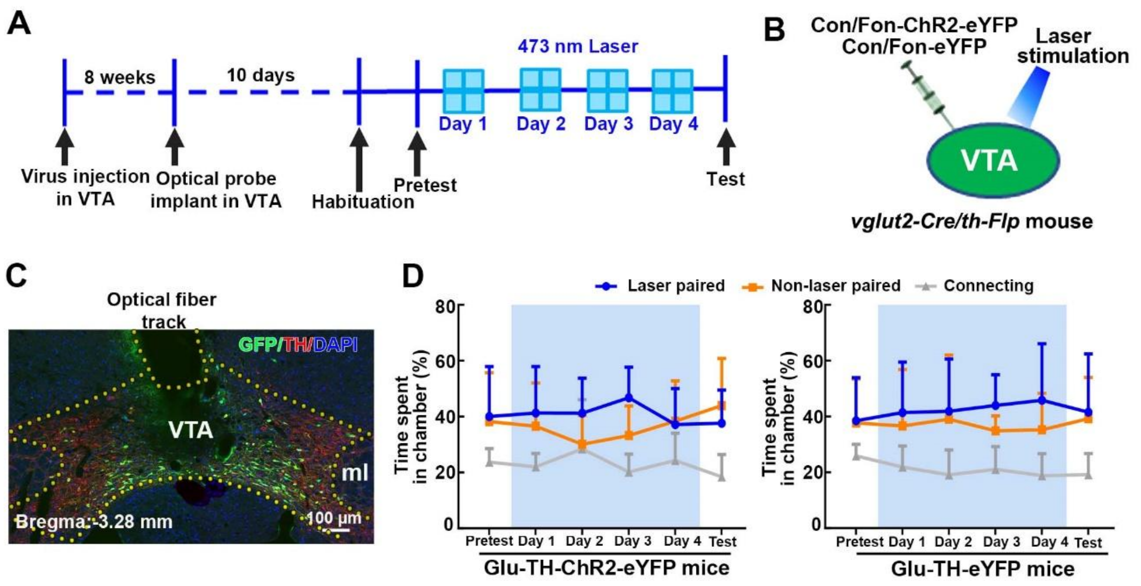

Supplementary figure 12. Photostimulation of VTA$^{glutamate\text{-}dopamine}$ neurons does not induce reward or aversion. A. Timeline for behavioral testing. **B.** VTA injection of Con/Fon viral vectors in *vglut2-Cre/th-Flp* mice (to target VTA$^{glutamate\text{-}dopamine}$ neurons) and VTA photostimulation. **C.** Detection of eYFP (green), TH (red), and DAPI (blue) expression within the VTA, and optical fiber placement. **D.** Glu-TH-ChR2-eYFP mice (n = 8) and Glu-TH-eYFP control mice (n = 7) spent similar time in the laser paired and non-laser paired chambers in the presence or absence of VTA photostimulation (chamber x day x group: $F_{(10,130)} = 0.57$; $p = 0.84$). Data are shown as mean ± SEM. Three-way ANOVA test. Source data are provided as a Source Data file.

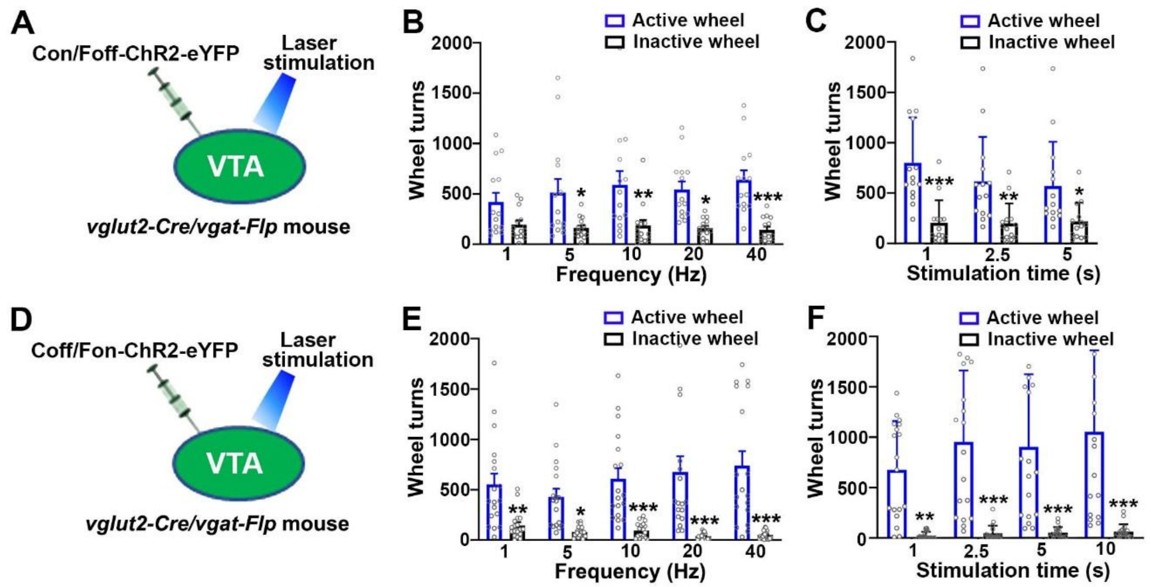

Supplementary figure 13. Photostimulation of VTA$^{glutamate\text{-}only}$ neurons at different parameters supports optical intracranial self-stimulation (oICSS), while photostimulation of VTA$^{GABA\text{-}only}$ neurons at different parameters induces oICSS avoidance. A. VTA injection of Con/Foff-ChR2 viral vector in *vglut2-Cre/vgat-Flp* mice (to target VTA$^{glutamate\text{-}only}$ neurons) and VTA photostimulation. **B.** Glu-only-ChR2-eYFP mice (n = 7) rotated the active wheel significantly more times than the inactive wheel at the tested frequencies, except at 1 Hz (wheel: $F_{(1,65)} = 61.78$, p = 0.00001), without a dose-effect of the frequencies used (wheel x frequency: $F_{(4,65)} = 0.88$, p = 0.48). **C.** Glu-only-ChR2-eYFP mice (n = 7) rotated the active wheel significantly more than the inactive wheel at all the tested stimulation times (wheel: $F_{(1,39)} = 44.74$, p = 0.00001), without a dose-effect of the stimulation times used (wheel x time: $F_{(2,39)} = 1.22$, p = 0.30). **D.** VTA injection of Coff/Fon-ChR2 viral vector in *vglut2-Cre/vgat-Flp* mice (to target VTA$^{GABA\text{-}only}$ neurons) and VTA photostimulation. **E.** GABA-only-ChR2-eYFP mice (n = 9) rotated the active wheel to stop photostimulation significantly more times than the inactive wheel at all the tested frequencies (wheel: $F_{(1,85)} = 100.25$, p = 0.00001), without a dose-effect of the frequencies used (wheel x frequency: $F_{(4,85)} = 1.32$, p = 0.27). **F.** GABA-only-ChR2-eYFP mice (n = 9) rotated the active wheel significantly more times than the inactive wheel to stop photostimulation at all the tested stimulation times (wheels: $F_{(1,68)} = 113.60$, p = 0.00001), without a dose-effect of the stimulation times used (wheel x time: $F_{(3,68)} = 0.82$, p = 0.49). Data are shown as mean ± SEM. Two-way ANOVA with Tukey HSD post hoc test. * p < 0.05, ** p < 0.01, *** p < 0.001, against active wheel. Source data are provided as a Source Data file.

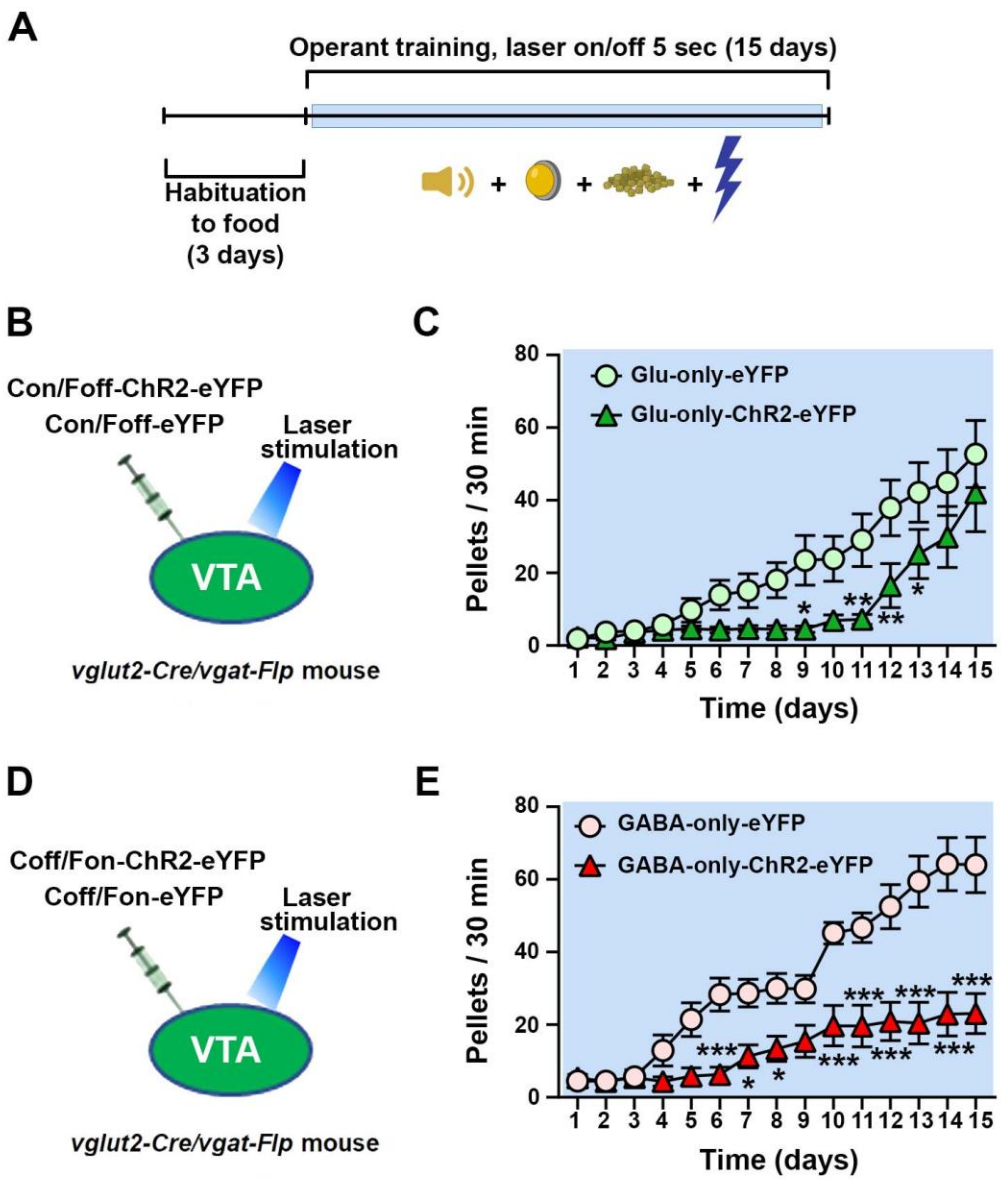

Supplementary figure 14. Photostimulation of VTA$^{glutamate\text{-}only}$ neurons delays learning of food self-administration, while photostimulation of VTA$^{GABA\text{-}only}$ neurons impairs learning of food self-administration. A. Timeline of food self-administration, showing the operant training with cues, food, and laser administration (created with BioRender. Barbano, F. (2025) https://BioRender.com/7ec597z). **B.** VTA injection of Con/Foff viral vectors in *vglut2-Cre/vgat-Flp* mice (to target VTA$^{glutamate\text{-}only}$ neurons) and VTA photostimulation. **C.** Photostimulation of VTA$^{glutamate\text{-}only}$ neurons resulted in a delay of several days for Glu-only-ChR2-eYFP mice (n = 10) to obtain equivalent number of food pellets when compared to Glu-only-eYFP control mice (n = 11, group x day: $F_{(14,266)} = 1.80$; $p = 0.04$). **D.** VTA injection of Coff/Fon viral vectors in *vglut2-Cre/vgat-Flp* mice (to target VTA$^{GABA\text{-}only}$ neurons) and VTA photostimulation. **E.** Photostimulation of VTA$^{GABA\text{-}only}$ neurons resulted in a significant reduction in the number of food pellets obtained by GABA-only-ChR2-eYFP mice (n = 11) when compared to GABA-only-eYFP control mice (n = 10, group x day: $F_{(14,266)} = 11.23$; $p < 0.00001$). Light-blue rectangles indicate VTA photostimulation. Data are shown as mean ± SEM. Two-way ANOVA with Tukey HSD post hoc test. * $p < 0.05$, ** $p < 0.01$, *** $p < 0.001$, against eYFP control mice. Source data are provided as a Source Data file.

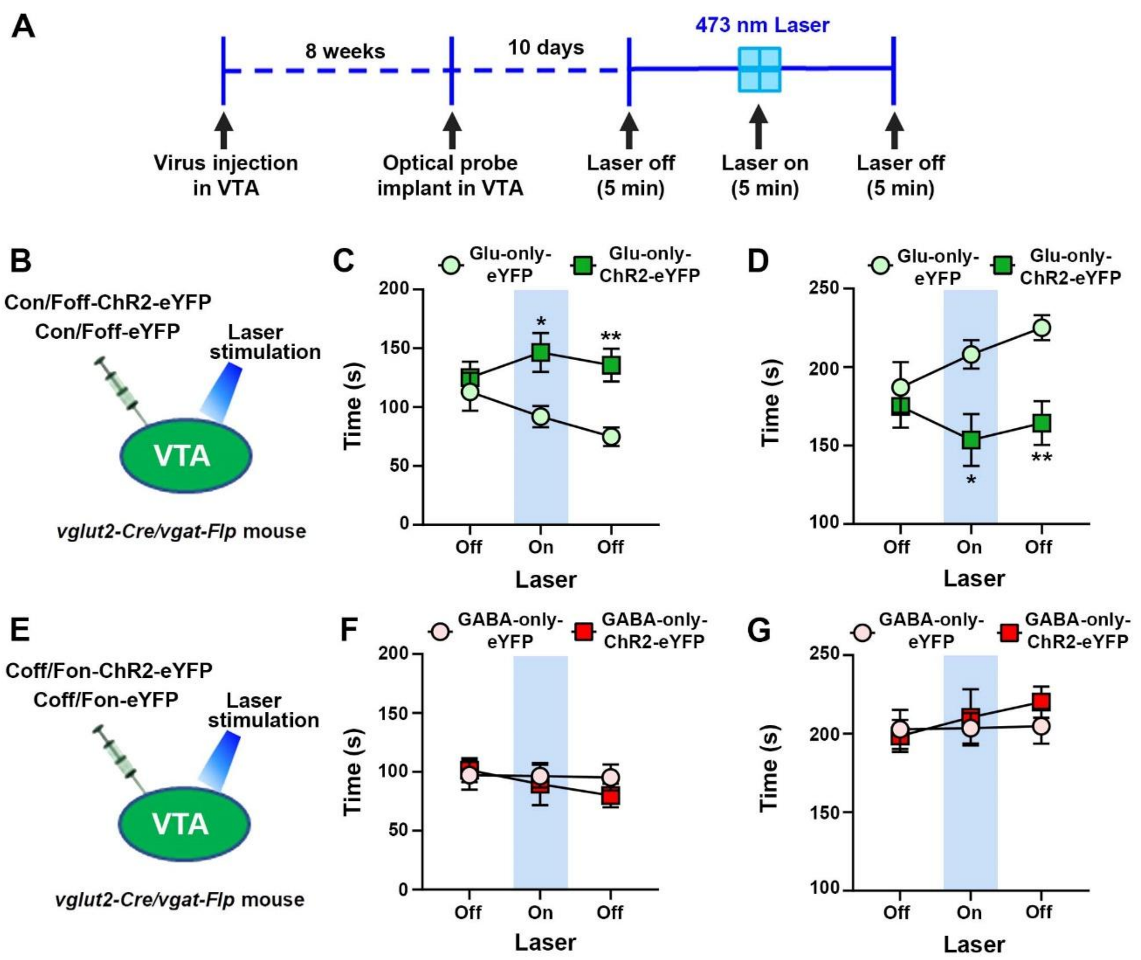

Supplementary figure 15. Photostimulation of VTA$^{glutamate\text{-}only}$ neurons decreases anxiety-like behaviors, while photostimulation of VTA$^{GABA\text{-}only}$ neurons does not modify anxiety-like behaviors. A. Timeline for elevated plus maze tests. **B.** VTA injection of Con/Foff viral vectors in *vglut2-Cre/vgat-Flp* mice (to target VTA$^{glutamate\text{-}only}$ neurons) and VTA photostimulation. **C-D.** Photostimulation of VTA$^{glutamate\text{-}only}$ neurons tended to increase the total time that Glu-only-ChR2-eYFP mice (n = 20) spent in the open arms (C; group x phase: $F_{(2,56)} = 2.81$; $p = 0.07$) and to decrease the total time that they spent in the closed arms (D; group x phase: $F_{(2,58)} = 2.81$; $p = 0.07$) of an elevated plus maze, when compared to Glu-only-eYFP control mice (n = 10). **E.** VTA injection of Coff/Fon viral vectors in *vglut2-Cre/vgat-Flp* mice (to target VTA$^{GABA\text{-}only}$ neurons) and VTA photostimulation. **F-G.** Photostimulation of VTA$^{GABA\text{-}only}$ neurons did not modify the total time that GABA-only-ChR2-eYFP mice (n = 11) spent in the open (F; group x phase: $F_{(2,40)} = 0.46$; $p = 0.63$) or closed (G; group x phase: $F_{(2,40)} = 0.46$; $p = 0.63$) arms of an elevated plus maze, when compared to GABA-only-eYFP control mice (n = 11). Light-blue rectangles indicate VTA photostimulation. Data are shown as mean ± SEM. Two-way ANOVA with Tukey HSD post hoc test. * $p < 0.05$, ** $p < 0.01$, against eYFP control mice. Source data are provided as a Source Data file.

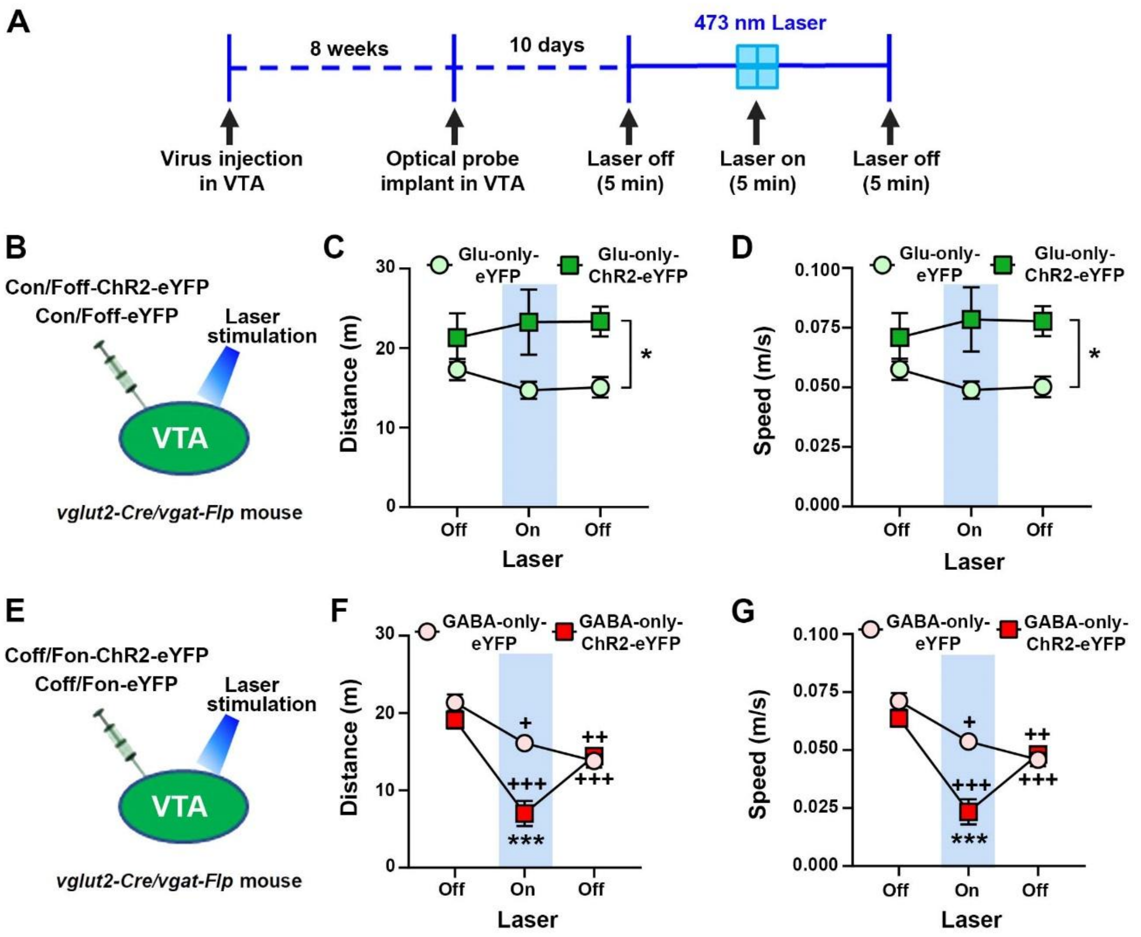

Supplementary figure 16. Photostimulation of VTA$^{\text{glutamate-only}}$ neurons does not modify locomotion, while photostimulation of VTA$^{\text{GABA-only}}$ neurons decreases locomotion. A. Timeline for open field tests. **B.** VTA injection of Con/Foff viral vectors in *vglut2-Cre/vgat-Flp* mice (to target VTA$^{\text{glutamate-only}}$ neurons) and VTA photostimulation. **C-D.** Photostimulation of VTA$^{\text{glutamate-only}}$ neurons did not modify the total distance travelled by mice (C, group x phase: $F(2,38) = 1.80$; $p = 0.19$) or their average speed (D**,** group x phase: $F(2,38) = 1.92$; $p = 0.16$) in an open field arena (Glu-only-ChR2-eYFP, n = 8; Glu-only-eYFP, n = 13). **E.** VTA injection of Coff/Fon viral vectors in *vglut2-Cre/vgat-Flp* mice (to target VTA$^{\text{GABA-only}}$ neurons) and VTA photostimulation. **F-G.** Photostimulation of VTA$^{\text{GABA-only}}$ neurons decreased the total distance travelled by GABA-only-ChR2-eYFP mice (F, n = 20, group x phase: $F_{(2,58)} = 11.81$; $p = 0.00005$) and their average speed (G, group x phase: $F_{(2,58)} = 11.82$; $p = 0.00005$) in an open field arena, when compared with GABA-only-eYFP control mice (n = 11). Light-blue rectangles indicate VTA photostimulation. Data are shown as mean ± SEM. Two-way ANOVA with Tukey HSD post hoc test. * $p < 0.05$, *** $p < 0.001$, against eYFP control mice; + $p < 0.05$, ++ $p < 0.01$, +++ $p < 0.001$, against the first period of laser off. Source data are provided as a Source Data file.

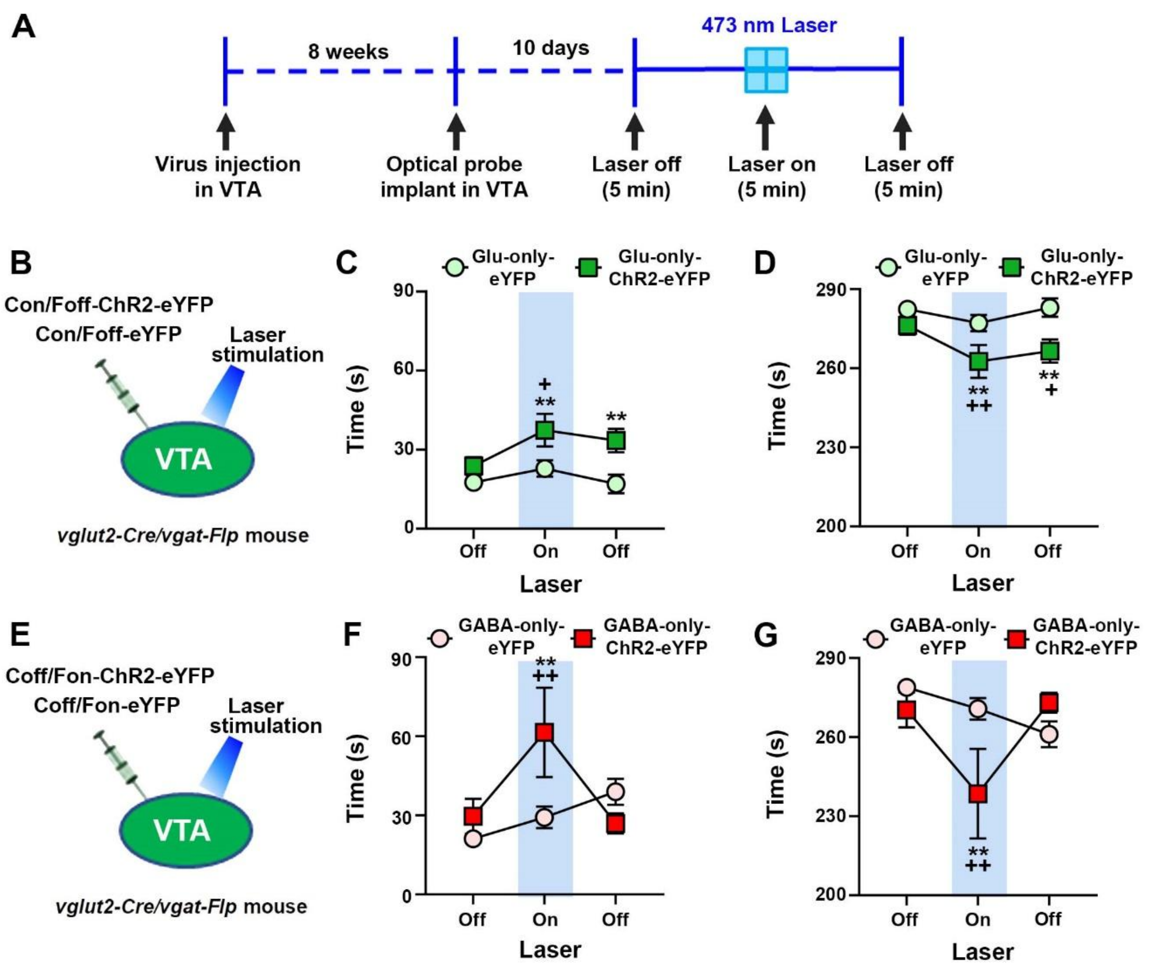

Supplementary figure 17. Photostimulation of VTA$^{glutamate\text{-}only}$ or VTA$^{GABA\text{-}only}$ neurons modulates anxiety-like behaviors. A. Timeline for the open field test. **B.** VTA injection of Con/Foff viral vectors in *vglut2-Cre/vgat-Flp* mice (to target VTA$^{glutamate\text{-}only}$ neurons) and VTA photostimulation**. C-D.** Photostimulation of VTA$^{glutamate\text{-}only}$ neurons tended to increase the total time that Glu-only-ChR2-eYFP mice (n = 14) spent in the center (C; group x phase: $F_{(2,50)} = 1.84$; $p = 0.17$) and to decrease the time they spent in the periphery (D; group x phase: $F_{(2,50)} = 1.84$; $p = 0.17$) of an open field arena, when compared to Glu-only-eYFP control mice (n = 13). **E.** VTA injection of Coff/Fon viral vectors in *vglut2-Cre/vgat-Flp* mice (to target VTA$^{GABA\text{-}only}$ neurons) and VTA photostimulation**. F-G.** Photostimulation of VTA$^{GABA\text{-}only}$ neurons increased the total time that GABA-only-ChR2-eYFP mice (n = 11) spent in the center (F; group x phase: $F_{(2,40)} = 5.72$; $p = 0.007$) and decreased the total time that they spent in the periphery (G; group x phase: $F_{(2,40)} = 5.72$; $p = 0.007$) of an open field arena, when compared to GABA-only-eYFP control mice (n = 11). Light-blue rectangles indicate VTA photostimulation. Data are shown as mean ± SEM. Two-way ANOVA with Tukey HSD post hoc test. ** $p < 0.01$, against eYFP control mice; + $p < 0.05$, ++ $p < 0.01$, against the first period of laser off. Source data are provided as a Source Data file.

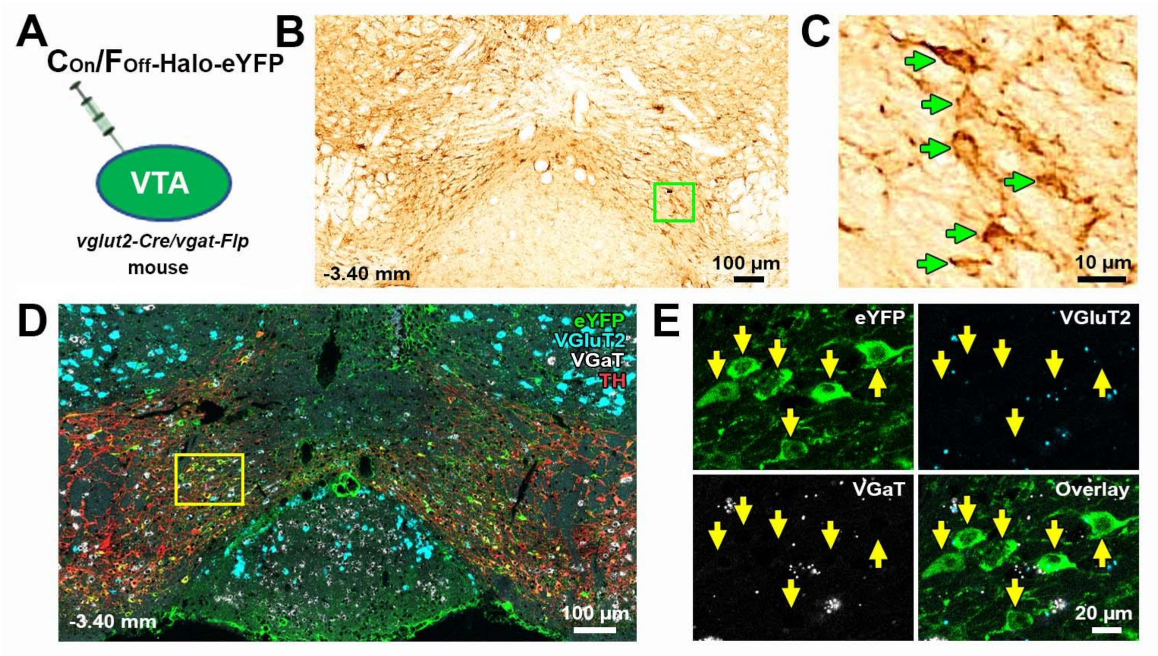

Supplementary figure 18. Targeting of VTA$^{glutamate\text{-}only}$ neurons with INTRSECT inhibitory viral vectors. A. VTA injection of Con/Foff-Halo-eYFP viral vector in *vglut2-Cre/vgat-Flp* mice (to target VTA$^{glutamate\text{-}only}$ neurons). **B-C.** VTA cellular expression of eYFP induced by injection of Con/Foff-Halo-eYFP viral vector, low (B) and high (C) magnification. **D-E.** Lack of detection of VGluT2 or VGaT mRNAs in eYFP expressing neurons, low (D) and high (E) magnification.

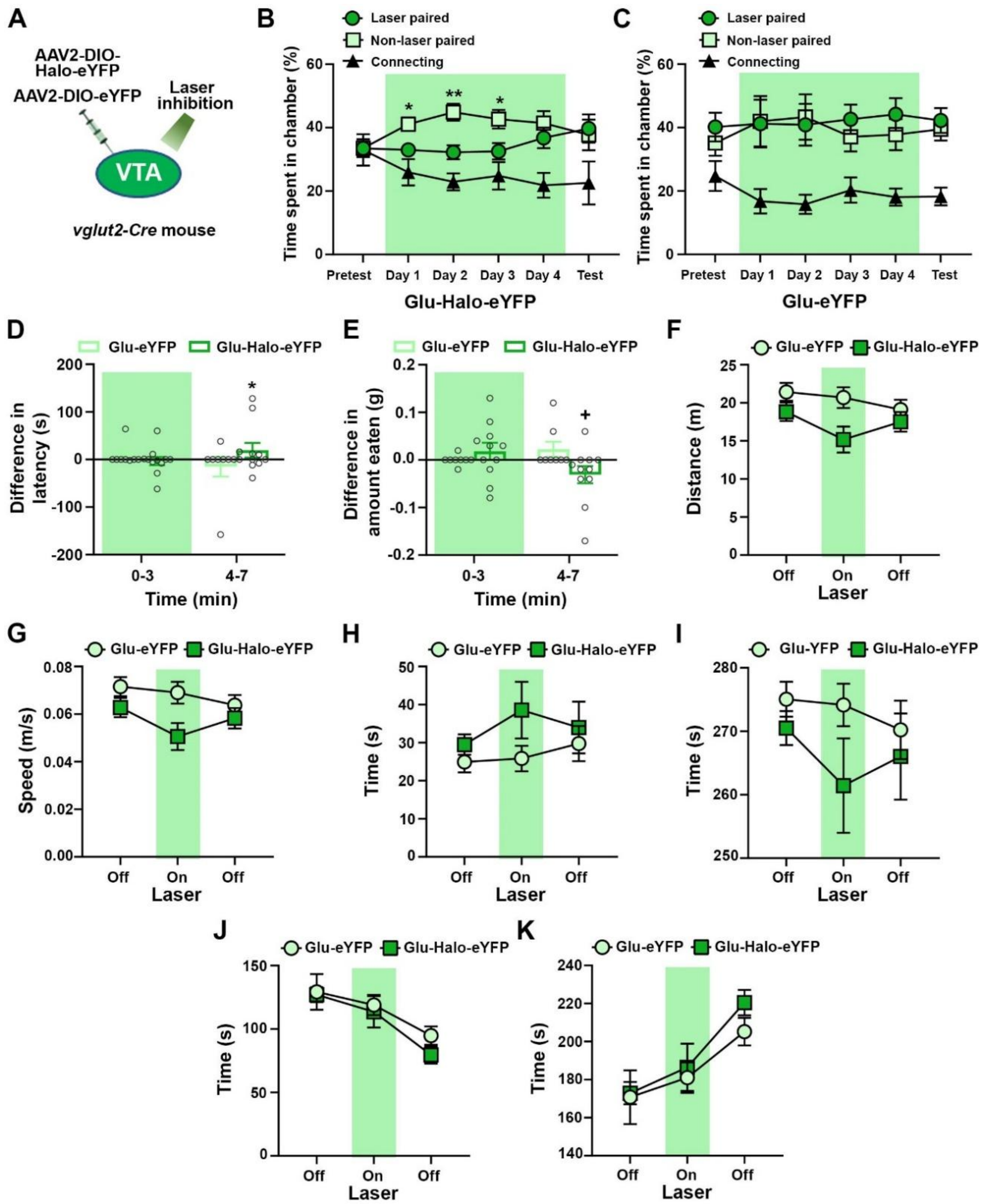

Supplementary figure 19. Photoinhibition of VTA^{glutamate} neurons is aversive and increases food intake. **A.** VTA injection of AAV2-DIO-Halo-eYFP or AAV2-DIO-eYFP viral vectors in *vglut2-Cre* mice (to target VTA^{glutamate} neurons) and VTA photoinhibition. **B-C.** Glu-Halo-eYFP mice (B, n = 11; chamber x day: $F_{(10,100)} = 2.07$; p = 0.03), but not Glu-eYFP control mice (C, n = 9; chamber x day: $F_{(10,70)} = 0.35$; p = 0.96), spent significantly less time in the laser-paired chamber during most of the photoinhibition sessions without developing conditioned place aversion for the laser-paired chamber. **D.** Photoinhibition of VTA^{glutamate} neurons did not affect feeding initiation latency during laser trials (0-3 min), but latency was increased during no-laser trials (4-7 min) in Glu-Halo-eYFP mice (n = 11) compared to Glu-eYFP control mice (n = 8; group x phase: $F_{(1,17)} = 4.47$; p = 0.05). **E.** Photoinhibition of VTA^{glutamate} neurons did not affect the amount of food eaten during laser (0-3 min) or no-laser trials (4-7 min) in Glu-Halo-eYFP mice (n = 11) compared to Glu-eYFP control mice (n = 8). However, Glu-Halo-eYFP mice consumed more food in the presence of photoinhibition than in its absence (group x phase: $F_{(1,17)} = 6.42$; p = 0.02). **F-G.** Photoinhibition of VTA^{glutamate} neurons did not modify the total distance travelled by mice (F, group x phase: $F_{(2,34)} = 2.25$; p = 0.12) or their average speed (G**,** group x phase: $F_{(2,34)} = 2.26$; p = 0.12) in an open field arena (Glu-Halo-eYFP, n = 11, Glu-eYFP, n = 8). **H-I.** Photoinhibition of VTA^{glutamate} neurons did not change the total time that Glu-Halo-eYFP mice (n = 11) spent in the center (H; group x phase: $F_{(2,34)} = 0.71$; p = 0.50) or the periphery (I; group x phase: $F_{(2,34)} = 0.71$; p = 0.50) of an open field arena, when compared to Glu-eYFP control mice (n = 8). **J-K.** Photoinhibition of VTA^{glutamate} neurons did not change the total time that Glu-Halo-eYFP mice (n = 11) spent in the open (J; group x phase: $F_{(2,34)} = 0.47$; p = 0.63) or closed (K; group x phase: $F_{(2,34)} = 0.47$; p = 0.63) arms of an elevated plus maze, when compared to Glu-eYFP control mice (n = 8). Green rectangles indicate VTA photoinhibition. Data are presented as mean ± SEM. Two- or three-way ANOVA with Tukey HSD post hoc test. * p < 0.05, ** p < 0.01, against eYFP control mice (or against non-laser paired chamber in B); + p < 0.05, against the laser phase. Source data are provided as a Source Data file.

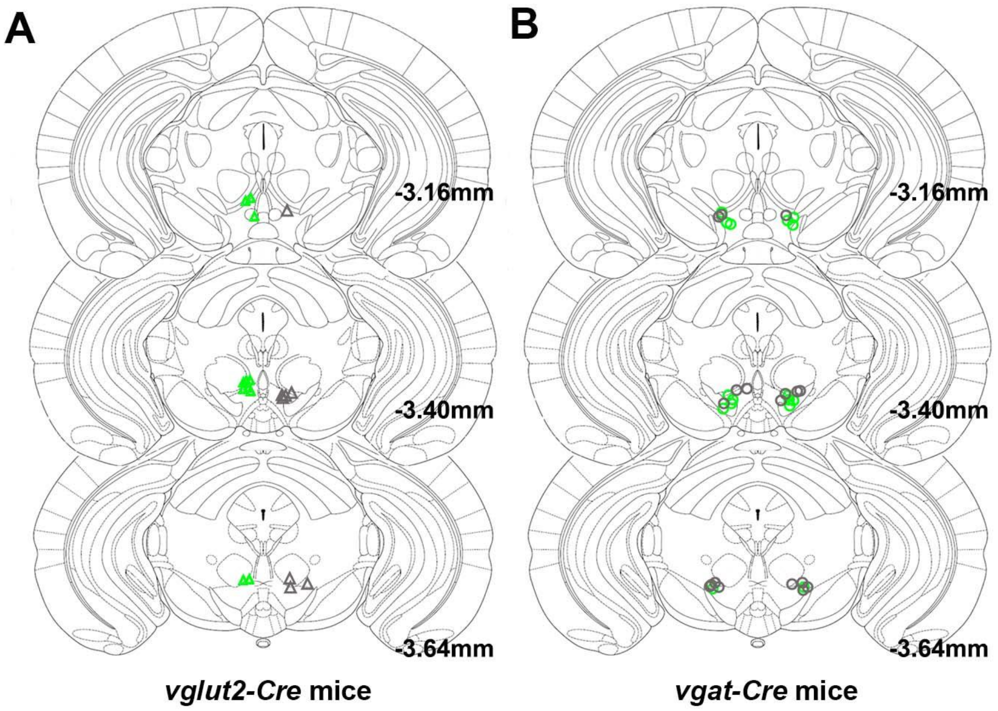

Supplementary figure 20. Optic fiber placements in the VTA for photoinhibition experiments. **A.** Optic fiber tips in the VTA of Glu-Halo-eYFP mice (n = 11, green triangles) and Glu-eYFP control mice (n = 8, grey triangles). **B.** Optical fiber tips in the VTA of GABA-Halo-eYFP mice (n = 8, green circles) and GABA-eYFP control mice (n = 8, grey circles).

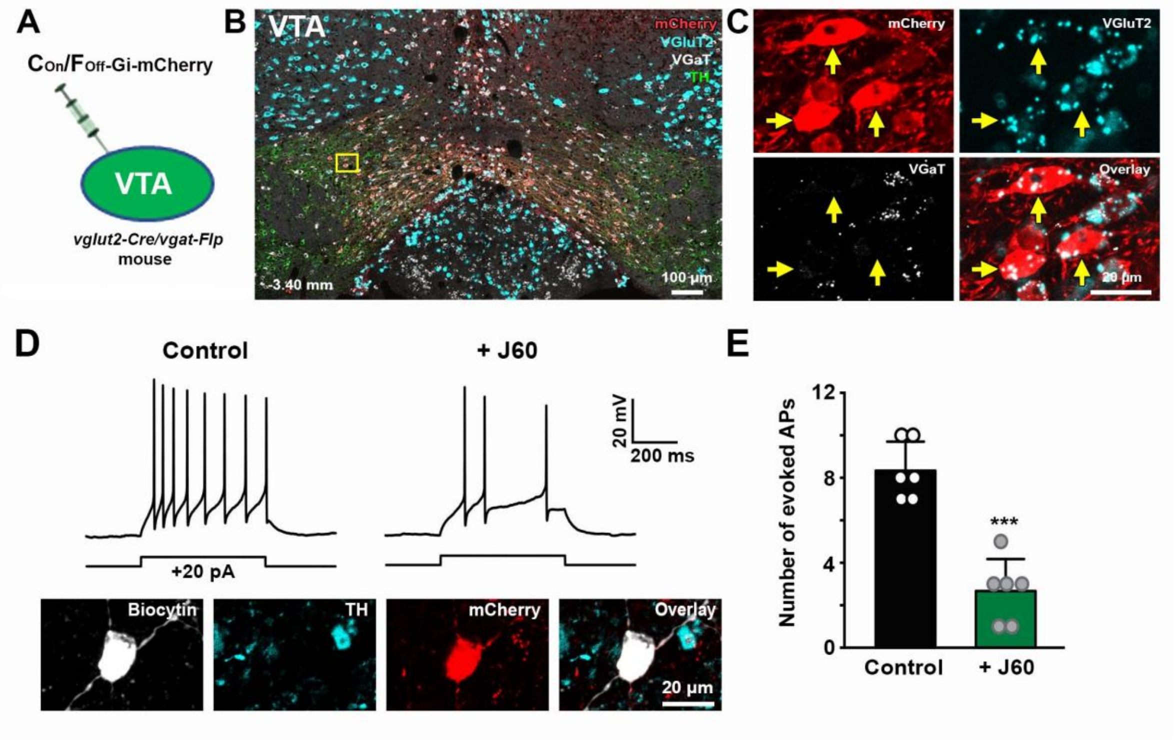

Supplementary figure 21. Targeting of VTA$^{glutamate\text{-}only}$ neurons with INTRSECT chemogenetic inhibitory viral vectors. A. VTA injection of Con/Foff-Gi-mCherry viral vector in *vglut2-Cre/vgat-Flp* mice (to target VTA$^{glutamate\text{-}only}$ neurons). **B-C.** Detection of transfected VTA neurons expressing mCherry, co-expressing VGluT2 mRNA, but lacking VGaT mRNA, low (B) and high (C) magnification. **D.** *Top panel.* Voltage responses of an mCherry-positive neuron to somatic current injection of 20 pA (600 ms duration) in control conditions (left) and after 10 min application of 10 µM J60 (right). *Bottom panel.* Brain horizontal section containing a VTA biocytin-filled cell (white) expressing mCherry (red) without TH (cyan). **E.** The number of evoked action potentials (APs) by current injection in mCherry-positive neurons in control conditions (8.33 ± 0.56) was significantly reduced after J60 administration (2.67 ± 0.61; n = 6, from 3 mice, $t_{(5)}$ = 11.46, p < 0.00001, paired t test). Data are shown as mean ± SEM. Source data are provided as a Source Data file.

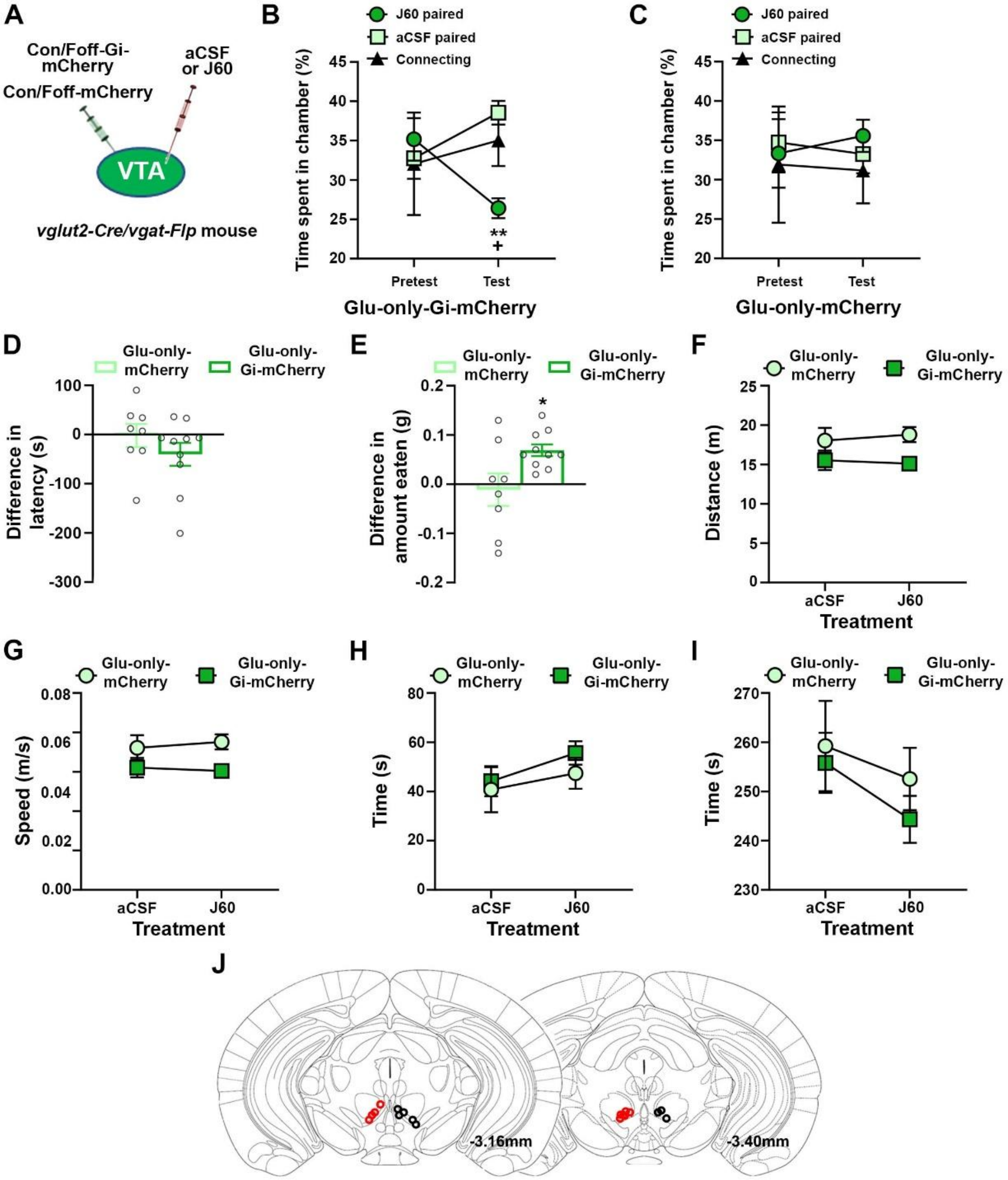

Supplementary figure 22. Chemogenetic inhibition of VTA$^{\text{glutamate-only}}$ neurons is aversive and increases food intake. A. VTA injection of Con/Foff viral vectors in *vglut2-Cre/vgat-Flp* mice (to target VTA$^{\text{glutamate-only}}$ neurons) and VTA microinjections of aCSF or J60**. B-C.** Glu-only-Gi-mCherry (B, n = 10; chamber x day: $F_{(2,18)} = 3.58$; p = 0.049), but not Glu-only-mCherry control mice (C, n = 8; chamber x day: $F_{(2,14)} = 0.21$; p = 0.81), developed a conditioned place aversion to the J60-paired chamber after the conditioning sessions. **D-E.** Chemogenetic inhibition of VTA$^{\text{glutamate-only}}$ neurons did not alter feeding initiation latency (D, $t_{(16)} = 1.13$; p = 0.27) but significantly increased the amount of food eaten (E, $t_{(16)} = -2.49$; p = 0.02) in Glu-only-Gi-mCherry mice (n = 10) when compared to Glu-only-mCherry control mice (n = 8). Differences in the amount eaten or the latency to start eating were calculated as the values obtained the day of J60 microinjection minus the values obtained the day of the aCSF microinjection. **F-G.** Chemogenetic inhibition of VTA$^{\text{glutamate-only}}$ neurons did not modify the total distance travelled by mice (F, group x phase: $F_{(1,16)} = 0.22$; p = 0.64) or their average speed (G**,** group x phase: $F_{(1,16)} = 0.23$; p = 0.64) in an open field arena (Glu-only-Gi-mCherry, n = 10, Glu-only-mCherry, n = 8). **H-I.** Chemogenetic inhibition of VTA$^{\text{glutamate-only}}$ neurons did not change the total time that Glu-only-Gi-mCherry mice (n = 10) spent in the center (H; group x phase: $F_{(1,16)} = 0.11$; p = 0.74) or the periphery (I; group x phase: $F_{(1,16)} = 0.11$; p = 0.74) of an open field arena, when compared to Glu-only-mCherry control mice (n = 8). **J.** Cannula tips in the VTA of Glu-only-Gi-mCherry mice (red circles) and Glu-only-mCherry control mice (black circles). Data are presented as mean ± SEM. Two-way ANOVA with Tukey HSD post hoc test or two-tailed t tests. * p < 0.05, ** p < 0.01, against Glu-only-mCherry mice (or against the aCSF paired chamber in B); + p < 0.05, against pretest. Source data are provided as a Source Data file.

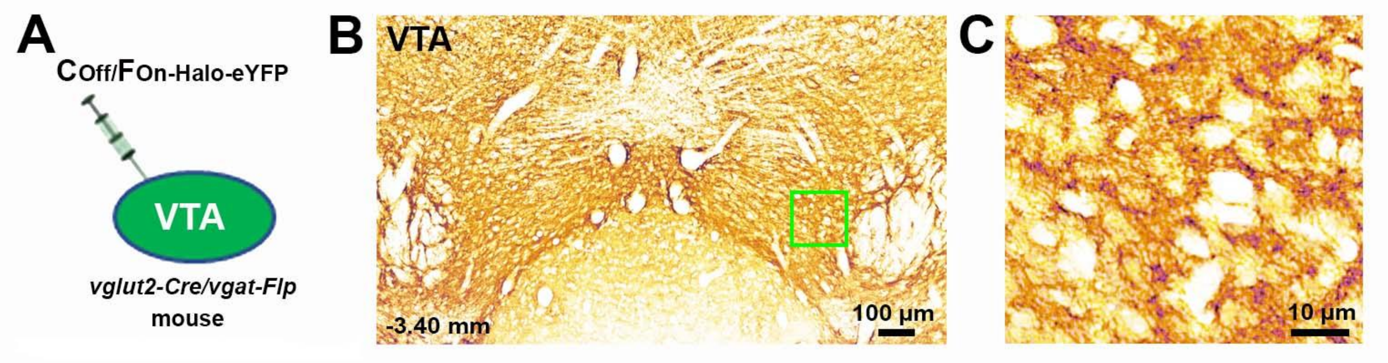

Supplementary figure 23. Targeting of VTA$^{GABA\text{-}only}$ neurons with INTRSECT inhibitory viral vectors. A. VTA injection of Coff/Fon-Halo-eYFP viral vector in *vglut2-Cre/vgat-Flp* mice (to target VTA$^{GABA\text{-}only}$ neurons). **B-C.** Lack of cellular detection on eYFP in VTA of mice that received intra-VTA injection of the Coff/Fon-Halo-eYFP viral vector, indicating lack of transfection. Low (B) and high (C) magnification.

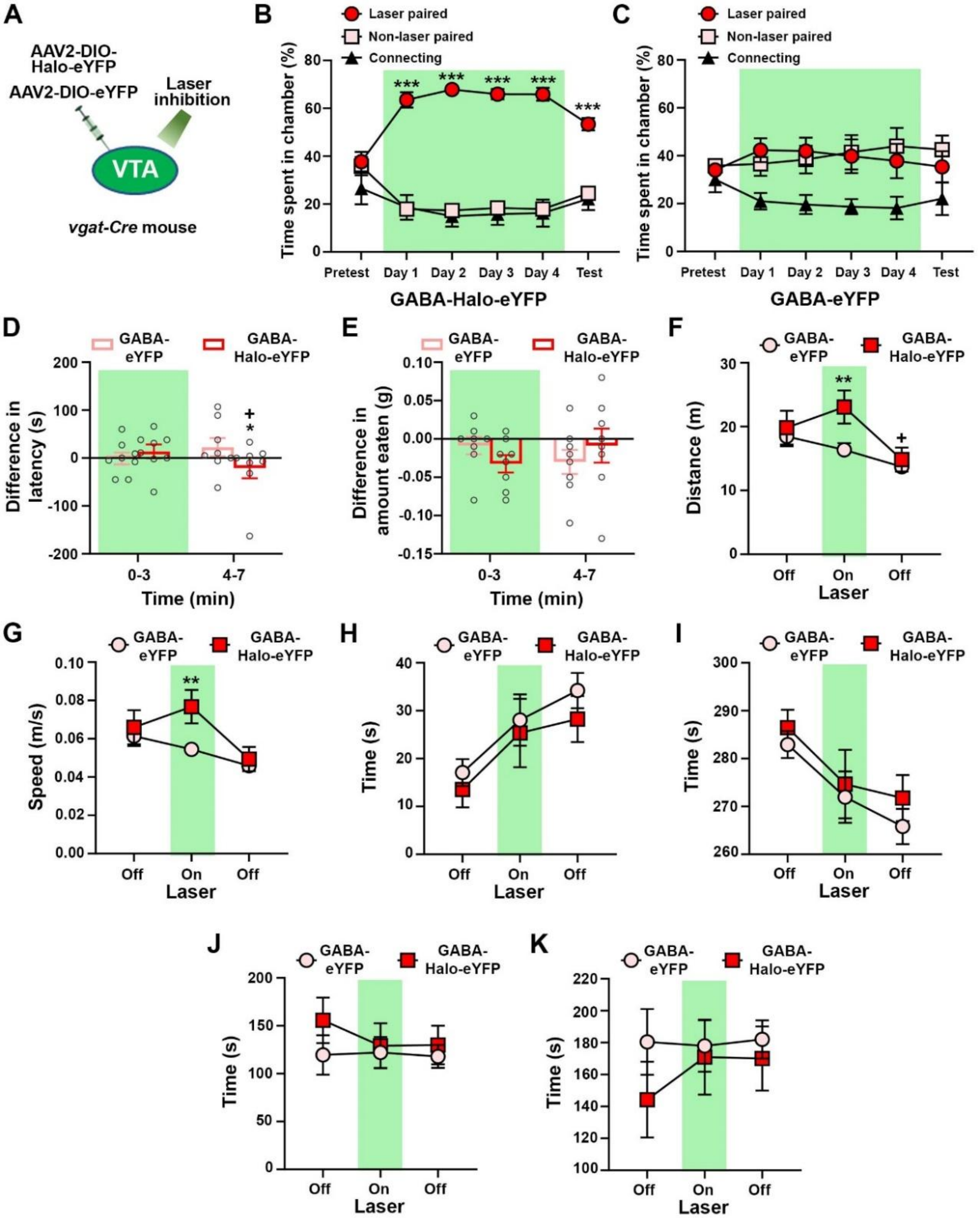

Supplementary figure 24. Photoinhibition of VTA^{GABA} neurons is rewarding and increases locomotor activity without modifying anxiety-like behaviors. **A.** VTA injection of AAV2-DIO-Halo-eYFP or AAV2-DIO-eYFP viral vectors in *vgat-Cre* mice (to target VTA^{GABA} neurons) and VTA photoinhibition. **B-C.** GABA-Halo-eYFP mice (B, n = 8; chamber x day: $F_{(10,70)} = 22.69$; p < 0.00001), but not GABA-eYFP control mice (C, n = 8; chamber x day: $F_{(10,70)} = 0.91$; p = 0.53), spent significantly more time in the laser-paired chamber during the photoinhibition sessions and also developed a conditioned place preference for the laser-paired chamber. **D-E.** Photoinhibition of VTA^{GABA} neurons did not affect feeding initiation latency during laser trials (0-3 min), but latency was decreased during no-laser trials (4-7 min) in GABA-Halo-eYFP mice (n = 8) compared to GABA-eYFP control mice (n = 8; group x phase: $F_{(1,14)} = 5.89$; p = 0.03). **E.** Photoinhibition of VTA^{GABA} neurons did not modify the amount of food eaten (group x phase: $F_{(1,14)} = 2.23$; p = 0.16) in GABA-Halo-eYFP mice (n = 8) when compared to GABA-eYFP control mice (n = 8). **F-G.** Photoinhibition of VTA^{GABA} neurons increased the total distance travelled (F, group x phase: $F_{(2,28)} = 3.74$; p = 0.04) and the average speed (G**,** group x phase: $F_{(2,28)} = 3.74$; p = 0.04) in an open field arena in GABA-Halo-eYFP mice (n = 8) but not in GABA-eYFP control mice (n = 8). **H-I.** Photoinhibition of VTA^{GABA} neurons did not change the total time that GABA-Halo-eYFP mice (n = 8) spent in the center (H; group x phase: $F_{(2,28)} = 0.12$; p = 0.89) or the periphery (I; group x phase: $F_{(2,28)} = 0.12$; p = 0.89) of an open field arena, when compared to GABA-eYFP control mice (n = 8). **J-K.** Photoinhibition of VTA^{GABA} neurons did not change the total time that GABA-Halo-eYFP mice (n = 8) spent in the open (J; group x phase: $F_{(2,28)} = 0.93$; p = 0.41) or closed (K; group x phase: $F_{(2,28)} = 0.93$; p = 0.41) arms of an elevated plus maze, when compared to GABA-eYFP control mice (n = 8). Green rectangles indicate VTA photoinhibition. Data are shown as mean ± SEM. Two- or three-way ANOVA with Tukey HSD post hoc test. * p < 0.05, ** p < 0.01, *** p < 0.001, against eYFP control mice (or against non-laser paired chamber in B); + p < 0.05, against the first period of laser off (or against the laser phase in D). Source data are provided as a Source Data file.

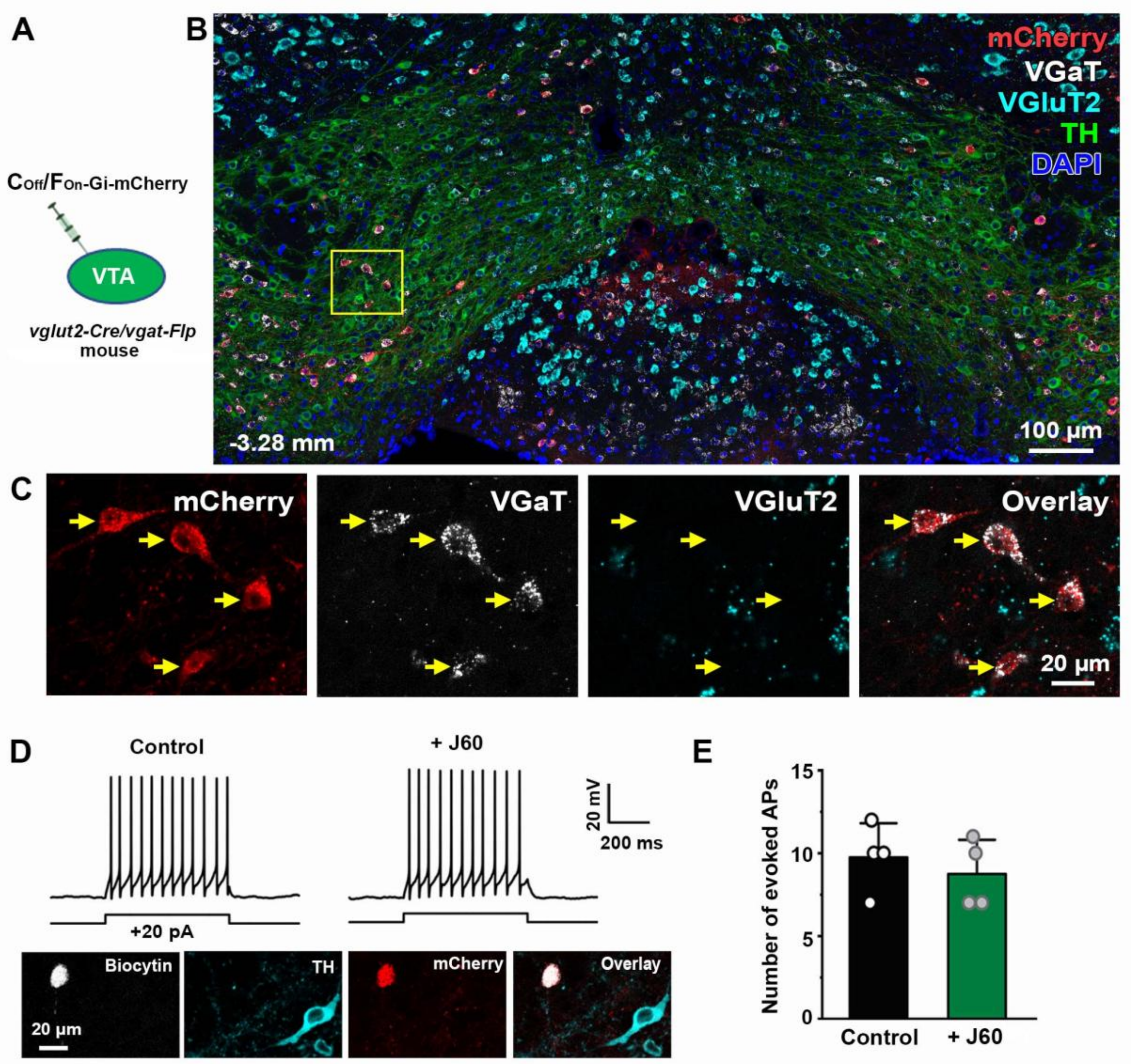

Supplementary figure 25. Targeting of VTA$^{\text{GABA-only}}$ neurons with INTRSECT chemogenetic inhibitory viral vectors. A. VTA injection of Coff/Fon-Gi-mCherry viral vector in *vglut2-Cre/vgat-Flp* mice (to target VTA$^{\text{GABA-only}}$ neurons). **B-C.** Detection of transfected VTA neurons expressing mCherry co-expressing VGaT mRNA, and lacking VGluT2 mRNA, low (B) and high (C) magnification. **D.** *Top panel.* Voltage responses of an mCherry-positive neuron to somatic current injection of 20 pA (600 ms duration) in control conditions (left) and after 10 min application of 10 µM J60 (right). *Bottom panel.* Brain coronal section containing a VTA biocytin-filled cell (white) expressing mCherry (red) without TH (cyan). **E.** The number of evoked action potentials (APs) by current injection in mCherry-positive neurons in baseline conditions (9.75 ± 1.03) and after J60 administration (8.75 ± 1.03) was not statistically different (n = 4, from 2 mice, $t_{(3)} = 1.41$, p = 0.25, paired t test). Data are shown as mean ± SEM. Source data are provided as a Source Data file.

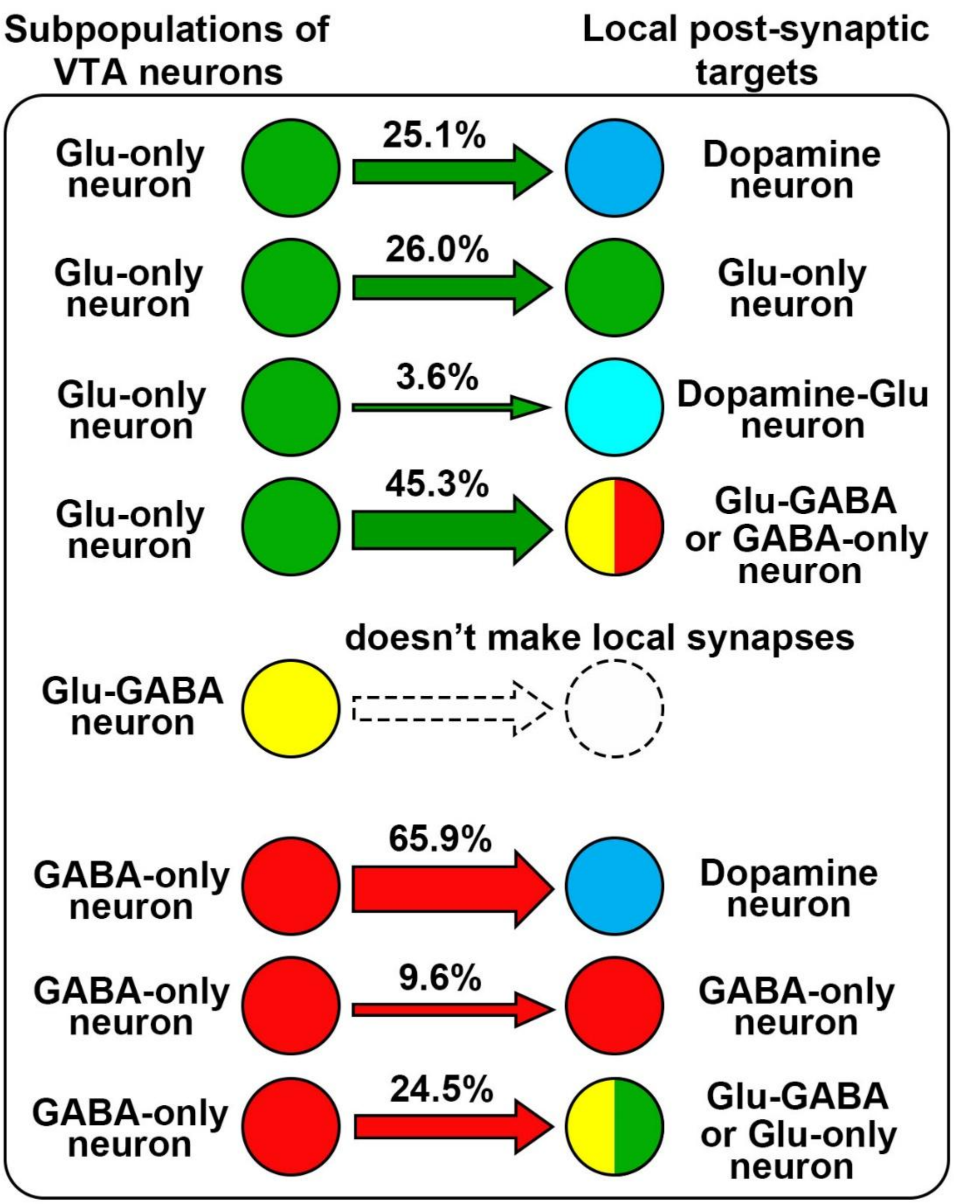

Supplementary Figure 26. VTA connectivity at the microcircuitry level. VTA$^{glutamate\text{-}only}$ neurons (green) establish asymmetric (excitatory) synapses at a similar frequency with both VTA^{dopamine} neurons (light blue) and VTA$^{glutamate\text{-}only}$ neurons, and rarely with dual VTA$^{dopamine\text{-}glutamate}$ neurons (cyan). VTA$^{glutamate\text{-}only}$ neurons frequently establish asymmetric synapses with a population of neurons integrated by VTA$^{glutamate\text{-}GABA}$ neurons (yellow) and VTA$^{GABA\text{-}only}$ neurons (red). Notably, dual VTA$^{glutamate\text{-}GABA}$ neurons do not establish local synapses.

VTA$^{GABA\text{-}only}$ neurons establish symmetric (inhibitory) synapses with the highest frequency with VTA^{dopamine} neurons and with low frequency with VTA$^{GABA\text{-}only}$ neurons, but with higher frequency with a population of neurons integrated by dual VTA$^{glutamate\text{-}GABA}$ neurons and VTA$^{glutamate\text{-}only}$ neurons.

Supplementary Tables

Supplementary table 1. Number of VTA axon terminals from VTA$^{glutamate\text{-}GABA}$, VTA$^{glutamate\text{-}only}$, and VTA$^{GABA\text{-}only}$ neurons

Bregma	VTA Area	[a]Axon terminals co-expressing eYFP, synaptophysin, VGluT2 and VGaT (n)		[b]Axon terminals co-expressing eYFP, synaptophysin and VGluT2 and lacking VGaT (n)		[c]Axon terminals co-expressing eYFP, synaptophysin, VGaT and lacking VGluT2 (n)	
			Total		Total		Total
-3.08 mm	Medial	64	121	1087	2802	539	1513
	Mediolateral	41		827		505	
	Lateral	16		888		469	
-3.40 mm	Medial	43	123	1126	3240	356	1195
	Mediolateral	46		1356		511	
	Lateral	34		758		328	
-3.64 mm	Medial	22	51	1072	3065	570	2261
	Mediolateral	14		983		818	
	Lateral	15		1010		873	
Total number of counted VTA axon terminals			**295**		**9107**		**4969**
Frequency of VTA identified axon terminals from local neurons			**2%**		**63%**		**35%**

[a]Expression of eYFP was driven in VTA$^{glutamate\text{-}GABA}$ neurons for quantification of their local axon terminals identified by the co-expression of eYFP, synaptophysin, VGluT2 and VGaT. Analysis of the rostro-caudal distribution of axon terminals within the medial, mediolateral and lateral aspects of the VTA.

[b] Expression of eYFP was driven in VTA$^{glutamate\text{-}only}$ neurons for quantification of their local axon terminals identified by the co-expression of eYFP, synaptophysin, and VGluT2. Analysis of the rostro-caudal distribution of axon terminals within the medial, mediolateral and lateral aspects of the VTA.

[c] Expression of eYFP was driven in VTA$^{GABA\text{-}only}$ neurons for quantification of their local axon terminals identified by the co-expression of eYFP, synaptophysin, and VGaT. Analysis of the rostro-caudal distribution of axon terminals within the medial, mediolateral and lateral aspects of the VTA.

Data were collected from 3 *vglut2-Cre/vgat-Flp* mice with intra-VTA injections of Con/Fon-ChR2-eYFP viral vector to drive the expression of eYFP in VTA$^{glutamate\text{-}GABA}$ neurons; 3 *vglut2-Cre/vgat-Flp* mice with intra-VTA injections of Con/Foff-ChR2-eYFP viral vector to drive the expression of eYFP in VTA$^{glutamate\text{-}only}$ neurons; and 3 *vglut2-Cre/vgat-Flp* mice with intra-VTA injections of Coff/Fon-ChR2-eYFP viral vector to drive the expression of eYFP in VTA$^{GABA\text{-}only}$ neurons. VTA mediolateral boundaries: medial VTA (0-0.25 mm), mediolateral VTA (0.25-0.50 mm), lateral VTA (> 0.50 mm).

Supplementary table 2. Number of axon terminals from VTA$^{glutamate\text{-}only}$ neurons making synapses on VTA neurons

Axon terminals from VTA$^{glutamate\text{-}only}$ neurons synapsing on different subpopulation of VTA neurons		
**Synapses within the medial VTA**	**Synapses within the lateral VTA**	**Total number of synapses**
Synapses on VTA$^{dopamine\text{-}only}$ neurons (n = 91) (91/454 = 19.6 ± 4.3%)	Synapses on VTA$^{dopamine\text{-}only}$ neurons (n = 110) (110/340 = 32.2 ± 5.8%)	Synapses on VTA$^{dopamine\text{-}only}$ neurons (n = 201) (201/794 = 25.1 ± 3.6%)
Synapses on VTA$^{glutamate\text{-}only}$ neurons (n = 158) (158/454 = 35.1 ± 6.4%)	Synapses on VTA$^{glutamate\text{-}only}$ neurons (n = 47) (47/340 = 14.0 ± 3.7%)	Synapses on VTA$^{glutamate\text{-}only}$ neurons (n = 205) (205/794 = 26.0 ± 5.2%)
Synapses on VTA$^{dopamine\text{-}glutamate}$ neurons (n = 14) (14/454 = 3.0 ± 1.4%)	Synapses on VTA$^{dopamine\text{-}glutamate}$ neurons (n = 14) (14/340 = 4.1 ± 1.9%)	Synapses on VTA$^{dopamine\text{-}glutamate}$ neurons (n = 28) (28/794 = 3.6 ± 0.7%)
Synapses on unlabeled neurons (n = 191) (191/454 = 42.3 ± 7.9%)	Synapses on unlabeled neurons (n = 169) (169/340 = 49.8 ± 4.1%)	Synapses on unlabeled neurons (n = 360) (360/794 = 45.3 ± 3.1%)
Total counted axon terminals in medial VTA (n = 454)	Total counted axon terminals in lateral VTA (n = 340)	Total counted axon terminals in VTA (n = 794)

Quantitative ultrastructural synaptic analysis of VTA from mice expressing eYFP in VTA$^{glutamate\text{-}only}$ neurons showing the frequency of axon terminals co-expressing eYFP and VGluT2, which synapsed on different classes of VTA neurons within the medial and lateral VTA. Data were collected from the VTA of 3 *vglut2-Cre/vgat-Flp* mice with intra-VTA injections of Con/Foff-ChR2-eYFP viral vector to drive the expression of eYFP in VTA$^{glutamate\text{-}only}$ neurons. VTA mediolateral boundaries: medial VTA (0-0.40 mm), lateral VTA (> 0.40 mm).

Supplementary table 3. Number of axon terminals from VTA$^{GABA\text{-}only}$ neurons making synapses on VTA neurons

Axon terminals from VTA$^{GABA\text{-}only}$ neurons synapsing on different subpopulation of VTA neurons		
**Synapses within the medial VTA**	**Synapses within the lateral VTA**	**Total number of synapses**
Synapses on VTA$^{dopamine\text{-}only}$ neurons (n = 187) (187/303 = 61.1 ± 3.9%)	Synapses on VTA$^{dopamine\text{-}only}$ neurons (n = 278) (278/402 = 68.9 ± 1.2%)	Synapses on VTA$^{dopamine\text{-}only}$ neurons (n = 465) (465/705 = 65.9 ± 1.1%)
Synapses on VTA$^{GABA\text{-}only}$ neurons (n = 24) (24/303 = 7.8 ± 0.6%)	Synapses on VTA$^{GABA\text{-}only}$ neurons (n = 43) (43/402 = 11.0 ± 1.7%)	Synapses on VTA$^{GABA\text{-}only}$ neurons (n = 67) (67/705 = 9.6 ± 0.9%)
Synapses on unlabeled neurons (n = 92) (92/303 = 31.1 ± 4.5%)	Synapses on unlabeled neurons (n = 81) (81/402 = 20.1 ± 0.6%)	Synapses on unlabeled neurons (n = 173) (173/705 = 24.5 ± 1.4%)
Total counted axon terminals in medial VTA (n = 303)	Total counted axon terminals in lateral VTA (n = 402)	Total counted axon terminals in VTA (n = 705)

Quantitative ultrastructural synaptic analysis of VTA from mice expressing eYFP in VTA$^{GABA\text{-}only}$ neurons showing the frequency of axon terminals co-expressing eYFP and VGaT, which synapsed on different classes of VTA neurons within the medial and lateral VTA. Data were collected from the VTA of 3 *vglut2-Cre/vgat-Flp* mice with intra-VTA injections of Coff/Fon-ChR2-eYFP viral vector to drive the expression of eYFP in VTA$^{GABA\text{-}only}$ neurons. VTA mediolateral boundaries: medial VTA (0-0.40 mm), lateral VTA (> 0.40 mm).

Supplementary table 4. VTA photostimulation of VTA$^{glutamate\text{-}only}$ neurons induced cFos expression in different subpopulations of VTA neurons.

**Mouse**	**cFos neurons co-expressing VGluT2 mRNA**	**cFos neurons co-expressing VGluT2 mRNA and VGaT mRNA**	**cFos neurons co-expressing TH protein**	**cFos neurons co-expressing TH protein and VGluT2 mRNA**	**cFos neurons co-expressing VGaT mRNA**	**cFos neurons lacking expression of VGluT2 mRNA, VGaT mRNA and TH protein**
[(a)]Glu-only-ChR2-eYFP mice						
Case 1	687	146	63	46	86	106
Case 2	690	119	80	50	147	112
Case 3	760	189	74	46	84	58
Mean ± SEM (n)	**712.3 ± 23.8 (n = 2137)**	**151.3 ± 20.4 (n = 454)**	**72.3 ± 4.9 (n = 217)**	**47.3 ± 1.3 (n = 142)**	**105.7 ± 20.7 (n = 317)**	**92.0 ± 17.1 (n = 276)**
[(b)]Glu-only-eYFP mice						
Case 1	101	23	10	25	58	74
Case 2	79	12	3	5	24	54
Case 3	47	16	13	8	23	35
Mean ± SEM (n)	**75.7 ± 15.7 (n = 227)**	**17.0 ± 3.2 (n = 51)**	**8.7 ± 2.9 (n = 26)**	**12.7 ± 6.2 (n = 38)**	**35.0 ± 11.5 (n = 105)**	**54.3 ± 11.3 (n = 163)**
[(c)]Ratio	**9.4**	**8.9**	**8.3**	**3.7**	**3.0**	**1.7**

[(a)]cFos neuronal detection after VTA photostimulation in mice expressing ChR2-eYFP in VTA$^{glutamate\text{-}only}$ neurons.
[(b)]cFos neuronal detection after VTA photostimulation in mice expressing eYFP in VTA$^{glutamate\text{-}only}$ neurons (control mice).
[(c)]Ratio of neurons expressing cFos between Glu-only-ChR2-eYFP and Glu-only-eYFP control mice for each subpopulation of VTA neurons.
Data were collected from 3 mice (15 sections per mouse).

Supplementary table 5. VTA photostimulation of VTA$^{GABA\text{-}only}$ neurons induced cFos expression in local neurons expressing VGaT mRNA

Mouse	cFos neurons co-expressing VGaT mRNA	cFos neurons co-expressing VGluT2 mRNA	cFos neurons co-expressing VGluT2 and VGaT mRNA	cFos neurons co-expressing TH protein	cFos neurons co-expressing TH protein and VGluT2 mRNA	cFos neurons lacking expression of VGluT2 mRNA, VGaT mRNA and TH protein
[(a)]GABA-only-ChR2-eYFP mice						
Case 1	421	115	37	3	0	82
Case 2	256	70	23	2	5	44
Case 3	339	101	17	1	5	54
Mean ± SEM (n)	**338.7 ± 47.6 (n = 1016)**	**95.3 ± 13.3 (n = 286)**	**25.7 ± 5.9 (n = 77)**	**2.0 ± 0.6 (n = 6)**	**3.3 ± 1.7 (n = 10)**	**60.0 ± 11.4 (n = 180)**
[(b)]GABA-only-eYFP mice						
Case 1	32	78	25	4	5	61
Case 2	14	50	23	3	10	20
Case 3	26	93	4	10	7	23
Mean ± SEM (n)	**24.0 ± 5.3 (n = 72)**	**73.7 ± 12.6 (n = 221)**	**17.3 ± 6.7 (n = 52)**	**5.7 ± 2.2 (n = 17)**	**7.3 ± 1.5 (n = 22)**	**34.7 ± 13.2 (n = 104)**
[(c)]Ratio	**14.1**	**1.3**	**1.5**	**-0.4**	**-0.5**	**1.7**

[(a)]cFos neuronal detection after VTA photostimulation in mice expressing ChR2-eYFP in VTA$^{GABA\text{-}only}$ neurons.
[(b)]cFos neuronal detection after VTA photostimulation in mice expressing eYFP in VTA$^{GABA\text{-}only}$ neurons (control mice).
[(c)]Ratio of neurons expressing cFos between GABA-only-ChR2-eYFP and GABA-only-eYFP mice for each subpopulation of VTA neurons.
Data were collected from 3 mice (15 sections per mouse).
