# Supplementary material for: VTA monosynaptic connections by local glutamate and GABA neurons and their distinct roles in behavior
Source: Nat Commun. 2025 Sep 26;16:8500. doi: 10.1038/s41467-025-63396-0 (PMC12475231; doi:10.1038/s41467-025-63396-0)
Supplement: Supplementary file 2 — Description of Additional Supplementary Files [file 41467_2025_63396_MOESM2_ESM.pdf]

Description of Additional Supplementary Files

File Name: Supplementary Movie 1
Description: VGluT2-axon terminal synapsing on a DA neuron. A 2D segmentation and 3D ultrastructural reconstruction of VTA local circuitry from serial scanning electron microscopic images showing that a VGluT2-axon terminal (co-expressing eYFP and VGluT2, green) from a VTAglutamate-only neuron established an asymmetric synapse (green arrow) on the soma from a VTAdopamine-only neuron (expressing TH, cyan).

File Name: Supplementary Movie 2
Description: VGluT2-axon terminal synapsing on an unlabeled neuron. A 2D segmentation and 3D ultrastructural reconstruction of VTA local circuitry from serial scanning electron microscopic images showing that a VGluT2-axon terminal (co-expressing eYFP and VGluT2, green) from a VTAglutamate-only neuron established an asymmetric synapse (green arrow) on the soma from a putative VTAglutamate-GABA neuron (without TH, eYFP, and mCherry signals, yellow).

File Name: Supplementary Movie 3
Description: VGaT-axon terminal synapsing on a DA neuron. A 2D segmentation and 3D ultrastructural reconstruction of VTA local circuitry from serial scanning electron microscopic images showing that a VGaT-axon terminal (co-expressing mCherry and VGaT, red) from a VTAGABA-only neuron established a symmetric synapse (red arrow) on the soma from a VTAdopamine-only neuron (expressing TH, cyan).

File Name: Supplementary Movie 4
Description: VGaT-axon terminal synapsing on an unlabeled neuron. A 2D segmentation and 3D ultrastructural reconstruction of VTA local circuitry from serial scanning electron microscopic images showing that a VGaT-axon terminal (co-expressing mCherry and VGaT, red) from a VTAGABA-only neuron established a symmetric synapse (red arrow) on the soma from a putative VTAglutamate-GABA neuron (without TH, eYFP, and mCherry signals, yellow).

File Name: Supplementary Movie 5
Description: VGluT2-axon terminal synapsing on a glutamate-GABA dendrite. A 2D segmentation and 3D ultrastructural reconstruction of VTA local circuitry from serial scanning electron microscopic images showing that a VGluT2- axon terminal (co-expressing eYFP and VGluT2, green) from a VTAglutamate-only neuron established an asymmetric synapse (green arrow) on the soma from a VTAglutamate-GABA dendrite (expressing mCherry, red).
