# Supplementary material for: VTA monosynaptic connections by local glutamate and GABA neurons and their distinct roles in behavior
Source: Nat Commun. 2025 Sep 26;16:8500. doi: 10.1038/s41467-025-63396-0 (PMC12475231; doi:10.1038/s41467-025-63396-0)
Supplement: Supplementary file 8 — Reporting Summary [file 41467_2025_63396_MOESM8_ESM.pdf]

nature portfolio

Corresponding author(s): Dr. Marisela Morales

Last updated by author(s): Jun 28, 2025

Reporting Summary

Nature Portfolio wishes to improve the reproducibility of the work that we publish. This form provides structure for consistency and transparency in reporting. For further information on Nature Portfolio policies, see our Editorial Policies and the Editorial Policy Checklist.

Statistics

For all statistical analyses, confirm that the following items are present in the figure legend, table legend, main text, or Methods section.

n/a	Confirmed	
☐	☒	The exact sample size (n) for each experimental group/condition, given as a discrete number and unit of measurement
☒	☐	A statement on whether measurements were taken from distinct samples or whether the same sample was measured repeatedly
☐	☒	The statistical test(s) used AND whether they are one- or two-sided<br>*Only common tests should be described solely by name; describe more complex techniques in the Methods section.*
☒	☐	A description of all covariates tested
☒	☐	A description of any assumptions or corrections, such as tests of normality and adjustment for multiple comparisons
☐	☒	A full description of the statistical parameters including central tendency (e.g. means) or other basic estimates (e.g. regression coefficient) AND variation (e.g. standard deviation) or associated estimates of uncertainty (e.g. confidence intervals)
☐	☒	For null hypothesis testing, the test statistic (e.g. F, t, r) with confidence intervals, effect sizes, degrees of freedom and P value noted<br>*Give P values as exact values whenever suitable.*
☒	☐	For Bayesian analysis, information on the choice of priors and Markov chain Monte Carlo settings
☒	☐	For hierarchical and complex designs, identification of the appropriate level for tests and full reporting of outcomes
☒	☐	Estimates of effect sizes (e.g. Cohen's d, Pearson's r), indicating how they were calculated

Our web collection on statistics for biologists contains articles on many of the points above.

Software and code

Policy information about availability of computer code

Data collection	Behavioral data was acquired with video tracking system Anymaze (Stoelting). Confocal microscope images were acquired with Zen software (Zeiss). Transmission electron microscopic images were collected using Gatan DigitalMicrograph software with Thermo Fisher Scientific FEI Tecnai G2 12. Serial electron microscopic images were collected using Atlas 5 software with Zeiss Sigma VP scanning electron microscope (SEM, Zeiss). Whole-cell voltage-clamp recordings were made using a MultiClamp 700B amplifier (Molecular Devices), low-pass filtered at 2 kHz and digitized at 10 kHz with pClamp 11.2 software (Molecular Devices).
Data analysis	Statistical analyses were performed using Statistica software (Cloud Software Group Inc.). The serial SEM images were aligned with the Amira software (Thermo Fisher Scientific). The serial confocal images and serial SEM images were correlated with the Imaris software (Bitplane Inc.) and then analyzed for 2D segmentation and 3D reconstruction movies with the Dragonfly software (Dragonfly).

For manuscripts utilizing custom algorithms or software that are central to the research but not yet described in published literature, software must be made available to editors and reviewers. We strongly encourage code deposition in a community repository (e.g. GitHub). See the Nature Portfolio guidelines for submitting code & software for further information.

Data

Policy information about availability of data

All manuscripts must include a data availability statement. This statement should provide the following information, where applicable:

- Accession codes, unique identifiers, or web links for publicly available datasets
- A description of any restrictions on data availability
- For clinical datasets or third party data, please ensure that the statement adheres to our policy

The source data generated in this study have been deposited in the Zenodo repository under DOI 10.5281/zenodo.15765068 (https://doi.org/10.5281/zenodo.15765069)

Research involving human participants, their data, or biological material

Policy information about studies with human participants or human data. See also policy information about sex, gender (identity/presentation), and sexual orientation and race, ethnicity and racism.

Reporting on sex and gender	Not applicable
Reporting on race, ethnicity, or other socially relevant groupings	Not applicable
Population characteristics	Not applicable
Recruitment	Not applicable
Ethics oversight	Not applicable

Note that full information on the approval of the study protocol must also be provided in the manuscript.

Field-specific reporting

Please select the one below that is the best fit for your research. If you are not sure, read the appropriate sections before making your selection.

☒ Life sciences ☐ Behavioural & social sciences ☐ Ecological, evolutionary & environmental sciences

For a reference copy of the document with all sections, see nature.com/documents/nr-reporting-summary-flat.pdf

Life sciences study design

All studies must disclose on these points even when the disclosure is negative.

Sample size	No statistical methods were used to predetermine sample size, but sample sizes are consistent with those reported in previous publications in the field and from our laboratory (Barker et al., 2023; Barbano et al., 2020; Root et al., 2020).
Data exclusions	In optogenetic experiments, mice with fiber tip placement outside of the target structure were excluded from the analysis.
Replication	All the experiments were successfully repeated three times to ensure reproducibility of the results and all attempts at replication were successful.
Randomization	Surgical and behavioral manipulations performed on each animal were randomly determined. In the case of behavioral experiments, the viral vector injected was determined randomly and counterbalanced across animals. Mice from each litter were randomly assigned into treatment groups.
Blinding	All experiments were conducted in a blind manner such that assays were conducted and analyzed without knowledge of the experimental group of the animal under study.

Reporting for specific materials, systems and methods

We require information from authors about some types of materials, experimental systems and methods used in many studies. Here, indicate whether each material, system or method listed is relevant to your study. If you are not sure if a list item applies to your research, read the appropriate section before selecting a response.

Materials & experimental systems

n/a	Involved in the study
	☒ Antibodies
☒	Eukaryotic cell lines
☒	Palaeontology and archaeology
	☒ Animals and other organisms
☒	Clinical data
☒	Dual use research of concern
☒	Plants

Methods

n/a	Involved in the study
☒	ChIP-seq
☒	Flow cytometry
☒	MRI-based neuroimaging

Antibodies

Antibodies used

Primary antibodies: mouse anti-GFP (1:500, 632381, Takara Bio USA, Inc.), rabbit anti-DsRed (1:500, 632496, Takara Bio USA, Inc.), mouse anti-tyrosine hydroxylase (1:500, MAB318, Millipore Sigma), mouse anti-synaptophysin (1:2000, MABN1193; Millipore Sigma), rabbit anti-VGaT (1:500, MSFR106160, Nittobo Medical), guinea pig anti-VGluT2 (1:500, MSFR106290, Nittobo Medical), rabbit anti-GFP (1:2000, MSFR101900, Nittobo Medical), guinea pig anti-VGaT (1:500, MSFR106160, Nittobo Medical, Japan), rabbit anti-VGluT2 (1:500, MSFR106310, Nittobo Medical).

Secondary antibodies: donkey anti-mouse Alexa Fluor 488 IgG (1:100, 715-545-151, Jackson ImmunoResearch Laboratories Inc.), Cy3 donkey anti-rabbit IgG (1:100, 711-165-152, Jackson ImmunoResearch Laboratories Inc.), DyLight 405 donkey anti-mouse IgG (1:100, 715-475-151, Jackson ImmunoResearch Laboratories Inc.), Cy5-Streptavidin (1:100; 016-170-084, Jackson ImmunoResearch Laboratories Inc.), donkey anti-rabbit Alexa Fluor 594 IgG (1:100, 711-585-152, Jackson ImmunoResearch Laboratories Inc.), donkey anti-guinea pig Alexa Fluor 647 IgG (1:100, 706-605-148, Jackson ImmunoResearch Laboratories Inc.), anti-guinea pig-IgG Fab′ fragment coupled to 1.4-nm gold (1:100, 2055-1ML, Nanoprobes), anti-mouse-IgG coupled to 1.4-nm gold (1:100, 2001-1ML, Nanoprobes), DyLight 405 donkey anti-guinea pig IgG (1:100, 706-475-1148, Jackson ImmunoResearch Laboratories Inc.), Alexa Fluor 647 donkey anti-rabbit IgG (1:100, 711-605-152, Jackson ImmunoResearch Laboratories Inc.), Alexa Fluor 647 donkey anti-mouse IgG (1:100, 715-605-151, Jackson ImmunoResearch Laboratories Inc.),Cy7 anti-mouse IgG (1:100, ab194808, Abcam).

Validation

All the primary antibodies used are commercially available and already validated (see manufacturer's websites). Mouse anti-GFP: validated by Western blot analysis using lysate made from a HEK 293 cell line stably expressing AcGFP1. A band of approximately 30 kDa corresponding to AcGFP1 was observed in the lane loaded with the AcGFP1 cell lysate. A band of this size was not detected in the lysate of untransfected HEK 293 cells. Rabbit anti-DsRed: validated by Western blot analysis. Lysate (10 μl; equivalent to 35,000 cells) from untransfected HEK 293 cells and lysates (10 μl; equivalent to 35,000 cells) from HEK 293 cells stably expressing DsRed-Express or AcGFP1 were resolved on a 12% SDS polyacrylamide gel and then transferred to a nitrocellulose membrane. The membrane was probed with the Living Colors DsRed Polyclonal Antibody (diluted 1:1,000), followed by secondary goat anti-rabbit antibody conjugated to horseradish peroxidase. The HRP signal was detected by chemiluminescence. A specific band of approximately 30–38 kDa was observed in the lane loaded with lysate from cells expressing DsRed-Express. No band in this molecular weight range was detected for the lysates of the untransfected HEK 293 cells or the cells expressing AcGFP1. Mouse anti-TH: validated by Western blot using lysate from PC12 cells or mouse brain lysates. Mouse anti-synaptophysin: validated by Western Blotting in rat brain tissue lysate. Rabbit anti-VGaT: validated by immunoblot detects a single protein band at 57 kDa as per manufacturer's 20221028 data sheet. Guinea pig anti-VGluT2: validated by immunoblot detects a single protein band at 60 kDa as per manufacturer's 20221028 data sheet. Rabbit anti-GFP: validated in a GFP transgenic mouse line, as per manufacturer's 20221027 data sheet (no staining observed in wild-type mice, reference: Takasaki et al., 2010, Eur. J. Neurosci.). Guinea pig anti-VGaT: validated by immunoblot detects a single protein band at 57 kDa as per manufacturer's 20221028 data sheet. Rabbit anti-VGluT2: validated by immunoblot detects a single protein band at 60 kDa as per manufacturer's 20221028 data sheet.

Animals and other research organisms

Policy information about studies involving animals; ARRIVE guidelines recommended for reporting animal research, and Sex and Gender in Research

Laboratory animals

Vglut2-IRES-Cre mice (JAX # 016963, The Jackson Laboratories, Bar Harbor, ME; on a mixed C57BL/6;FVB;129S6 genetic background) and slc32a1-IRES2-FlpO-D mice (vgat-FlpO-D, Jax # 031331, The Jackson Laboratories, on a mixed 129S6/SvEvTac x C57BL/6NCrl genetic background, deposited42) were crossed to produce the male and female vglut2-Cre/ vgat-Flp mice (20-30g) used in this study. vglut2-IRES-Cre and th-2A-Flpo mice (C57BL/6N-Thtm1Awar/Mmmh, in C57BL/6J background from the Mutant Mouse Resource and Research Centers, Davis, CA) were crossed to produce the vglut2-Cre/th-Flp male and female mice (20-30g) used in this study. vglut2-Cre mice (Slc17a6tm2(cre)Lowl/J, in C57BL/6J background from The Jackson Laboratories) and vgat-Cre mice (Slc32a1tm2(cre)Lowl/J, in C57BL/6J background from The Jackson Laboratories) were bred in the NIDA/IRP animal facility (20-30g) and were used in photoinhibition experiments. Groups of 2-5 mice were housed in a temperature- and humidity-controlled vivarium (at a constant temperature of 23 °C and 35-55% humidity) under a 12 h light/dark cycle (lights on at 7:00 am) with ad libitum access to food and water. Mice were 2-3 months old before any experimental manipulation.

Wild animals

The study did not involve wild animals.

Reporting on sex

Male and female mice were used in our study. Sex as a variable was analyzed in one of the experiments (Sup. Fig. 11) but, due to the lack of effect, was not further analyzed in the other experiments.

Field-collected samples

The study did not involve samples collected from the field.

Ethics oversight	Animal Care and Use Committee of the National Institute on Drug Abuse

Note that full information on the approval of the study protocol must also be provided in the manuscript.

Plants

Seed stocks	Not applicable
Novel plant genotypes	Not applicable
Authentication	Not applicable
